# Genomic continuity of Tai-Kadai-speaking populations from Southern China to Northern Thailand

**DOI:** 10.1186/s12915-025-02467-6

**Published:** 2025-12-22

**Authors:** Jatupol Kampuansai, Suwapat Sathupak, Wibhu Kutanan, Metawee Srikummool, Tanapon Seetaraso, Natcha Chaisoung

**Affiliations:** 1 Department of Biology, Faculty of Science, Chiang Mai University, Chiang Mai, Thailand; 2 Ph.D.’s Degree Program in Biology (International Program), Faculty of Science, Chiang Mai University, Chiang Mai, Thailand; 3 Department of Biology, Faculty of Science, Naresuan University, Phitsanulok, Thailand; 4 Department of Biochemistry, Faculty of Medical Science, Naresuan University, Phitsanulok, Thailand; 5 Center of Excellence in Medical Biotechnology, Faculty of Medical Science, Naresuan University, Phitsanulok, Thailand

**Keywords:** Tai-Kadai, Genetic ancestry, Migration, Greater Mekong Subregion, Ethnic group

## Abstract

**Background:**

Migration is a microevolutionary process that shapes cultural, societal, and genetic diversity in human populations. While previous genetic studies have examined the effects of migrations in several key areas of the world, there is a paucity of such studies in the upper Greater Mekong Subregion (GMS). The upper GMS, encompassing northern Thailand, Laos, Myanmar, and southern China, has been a major corridor for human migration and interaction between East and Southeast Asian populations for thousands of years.

**Results:**

We generated new genome-wide data for Tai-Kadai (TK)-speaking ethnic groups, namely Lue and Yong, from northern Thailand and integrated them with data from the upper GMS and across Asia. Our results highlight the genetic diversity among ethnic groups in the GMS, particularly the genetic continuity of TK migration from southern China to northern Thailand. The TK speakers in Thailand predominantly exhibit multiple ancestries from East Asia and Southeast Asia, with regional differentiations. The TK groups in northern Thailand primarily derive their genetic contributions from Dai-related communities, while northeastern Thai populations show a higher proportion of Lao-related ancestry. Those in central and southern Thailand display additional ancestries from other groups, such as Austroasiatic and South Asian populations. The genetic history of TK-speaking Lue populations illustrates the role of TK migration, founder effects, and historical resettlements in shaping genetic diversity.

**Conclusions:**

Overall, analyses of genome-wide data reveal that the genetic background of TK speakers in Thailand is predominantly of East Asian origin, with additional contribution from Southeast Asian populations. This pattern supports the idea of sustained migration from southern China into Thailand, particularly concentrated in the northern part. Our findings reinforce the historical continuity of TK movements across the upper GMS and provide new insights into the genetic and cultural transformations that have shaped present-day Thai populations.

**Supplementary Information:**

The online version contains supplementary material available at 10.1186/s12915-025-02467-6.

## Background

Migration, as a microevolutionary process, involves the transfer of individuals and their genetic variants between populations, playing a critical role in shaping genetic diversity, and through its demographic and historical consequences, can indirectly influence cultural, societal, and economic structures. As people migrate, they carry their sociocultural practices and genetic traits, which evolve through interactions with other populations and environmental factors. In recent decades, genomic evidence has been used to elucidate the migration of human beings in several key regions of the world, such as the massive migration of Indo-European speakers from Western Asia to Europe [1], maritime expansion of Austronesian (AN)-speaking peoples from East Asia to the Pacific [2, 3].

**Fig. 1:**
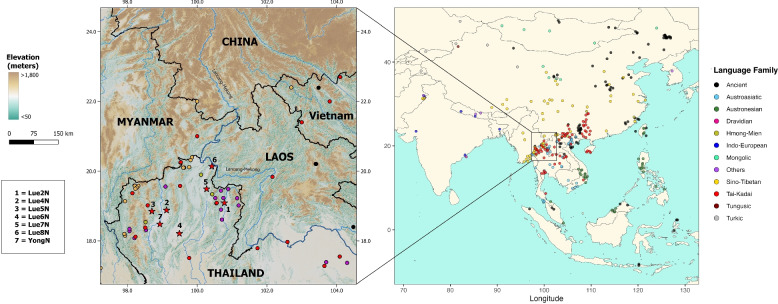
Map showing the locations of published datasets involved in the analysis. Each population is colored according to its language family, as indicated in the key on the right panel. The geographical map of the upper Greater Mekong Subregion is zoomed in on the left, showing terrain elevation and major rivers. The TK sampling locations, for which data were newly generated in this study, are represented by colored stars. The background map was created using QGIS 3.6.0 (http://www.qgis.org/) (left) and maps package in R software (right)

The upper Greater Mekong Subregion (GMS) is a key area for human migration in East and Southeast Asia, serving as a vital link between southern China and northern Thailand. This region includes the northern parts of the Mekong River basin, encompassing parts of southern China, Myanmar, Laos, Vietnam, and Thailand [4] (Fig. 1). With its diverse landscapes of mountains and river valleys, the upper GMS has been a center of human activity for thousands of years. Archeological findings, such as skeletal remains like Ban Rai rock shelter in Thailand and Tham Pa Ling (Monkey cave) in Laos, indicate human habitation in this area for over 10,000 years [5]. Its rich biodiversity and cultural heritage have made this region home to millions of people from diverse ethnic backgrounds.

Modern and ancient DNA studies highlight the genetic connections between prehistoric inhabitants and present-day populations of the upper GMS [6]. For example, genomic evidence from the log-coffin cultural community in Thailand shows admixture events involving Hoabinhian hunter-gatherers, Yangtze River farmers, and Yellow River farmers [7]. The shift from hunting and gathering to farming, driven by the spread of agricultural technologies around 4500 years ago, profoundly transformed the culture and demographics of the upper GMS. Genetic evidence suggests that this transition involved the migration of Austroasiatic (AA) speakers during the late Neolithic period, approximately 4500–3000 years ago. Later, Tai-Kadai (TK)-speaking populations began migrating into the region around 2000 years ago, with continued movements occurring up to 500 years ago [8, 9], leading to the fragmentation of AA groups and the expansion of TK communities.

The upper reaches of the GMS can be said to form the backbone of the TK world [10]. Today, the majority of people in the upper GMS, particularly in Thailand and Laos, are TK speakers, encompassing around 89% of total population [4]. The TK language family, which includes various local languages such as Thai, Lao, and Shan, is thought to have originated in the region between the Yangtze and Pearl rivers in China [11]. Linguistic similarities between TK-speaking populations in China and Thailand, such as shared vocabulary with similar meanings and pronunciations, reflect the cultural diffusion of the TK language. Although their accents have diverged over time and space, these populations still retain notable lexical overlap [12, 13]. Genetic evidence also reveals close relationships between TK speakers in Southeast Asia and their East Asian relatives [14, 15]. However, significant genetic diversity within TK populations reflects their dynamic migration history and interactions with local AA communities. The TK migration deeply influenced Thailand’s cultural and historical landscape, leading to the establishment of powerful kingdoms such as Lan Na and Sukhothai. These kingdoms played a central role in shaping Thai culture, including the spread of Buddhism, the creation of a distinct writing system, and the development of social and political institutions that continue to influence Thai society today [16].

Among the TK-speaking ethnic groups that migrated through the upper GMS, the Lue, or Xishuangbanna Dai people, stand out for their complex ethnohistorical background, shaped by diverse migration routes and wide geographic distribution. Their ancestral homeland can be traced to Yunnan Province in southern China, with a documented historical presence dating back to the twelfth century CE [17]. Over the past millennium, the Lue and their closely related Yong counterparts (Lue people from Yong City, now in Shan State, Myanmar) have undertaken transboundary migrations through Laos and Myanmar into Thailand in search of new agricultural settlements or to escape political instability. Today, they have resettled in various provinces across Thailand [17–19]. Although genomic data on the Lue have been included in previous studies, most focused on only two well-known and extensively studied Lue villages [15, 20]. This limited geographic and sample coverage has constrained our ability to fully capture the complexity of their population history, leaving an incomplete understanding of the broader genetic landscape and differentiation among TK-speaking communities in Thailand.

To address this knowledge gap, we collected comprehensive and representative samples from TK-speaking Lue and Yong populations and integrated them with publicly available genomic data from diverse ethnic groups. Recognizing the critical role of migration in shaping genetic diversity across the upper GMS, this study utilizes genome-wide single-nucleotide polymorphism (SNP) array analyses to investigate genetic continuity and population dynamics linked to the inland movement of TK speakers from southern China to northern Thailand. This approach offers valuable insights into the evolutionary history of populations in Thailand and its neighboring regions, shedding light on patterns of genetic exchange and the demographic development of Southeast Asia.

## Results

### Genetic affinity of the TK speakers among East and Southeast Asian populations

**Fig. 2:**
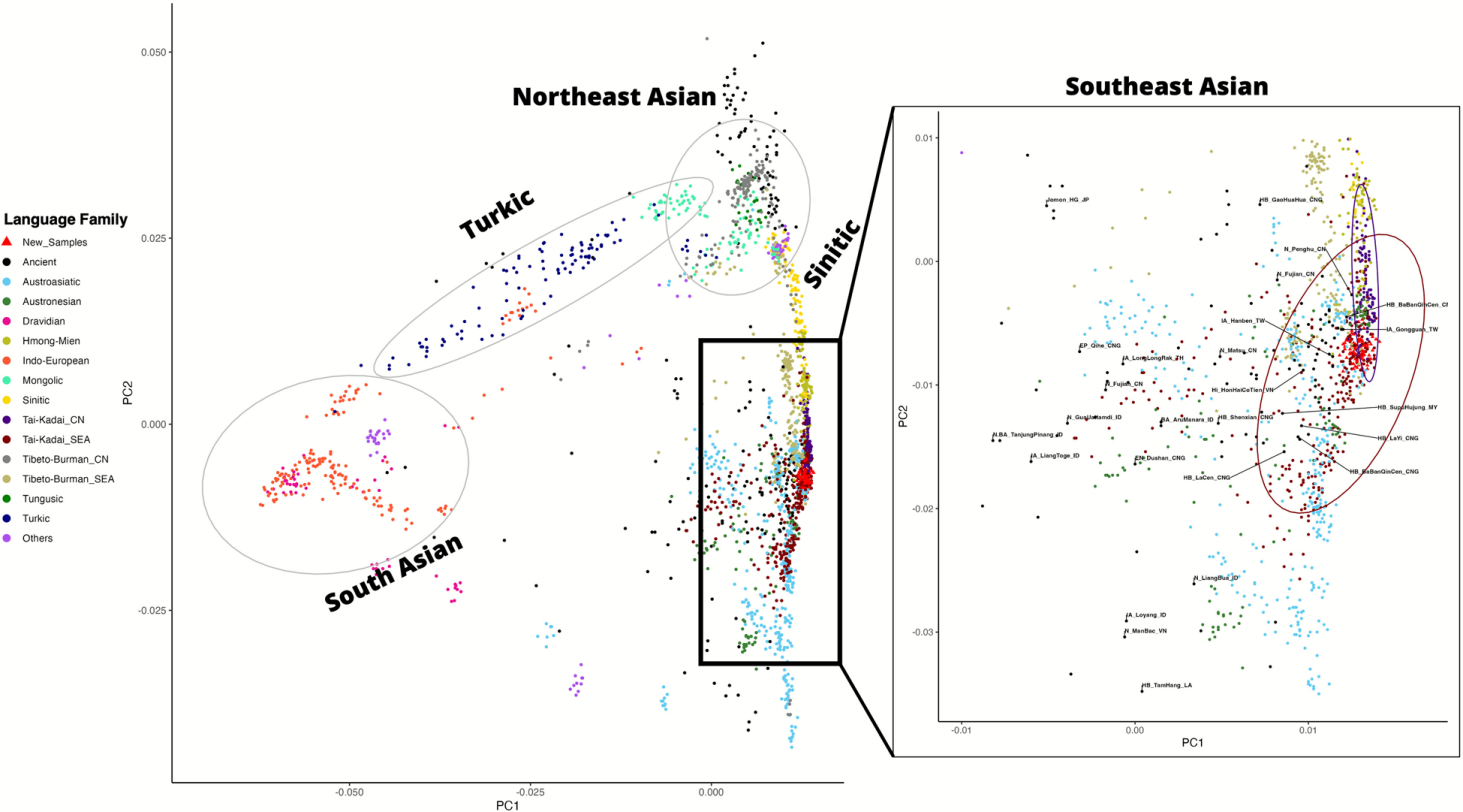
The principal component analysis (PCA) plot for the genome-wide SNP data of individuals from South, Northeast, and Southeast Asia is shown on the left. Each individual is colored by language family according to the key in the left panel. CN refers to populations in China while SEA refers to those in Southeast Asia. The plot focusing on the Southeast Asian populations is zoomed in to the right

We present newly generated genome-wide SNP data from 92 TK-speaking participants belonging to six Lue and one Yong ethnic groups from northern Thailand (Fig. 1 and Additional file 1: Table S1), using the Affymetrix Axiom Genome-Wide Human Origins array [21], which contains approximately 600,000 SNPs. These data were then combined and compared with previously published populations (Additional file 1: Table S2) to explore the genetic continuity and population dynamics of the TK-speaking ethnic groups across the upper GMS. The general patterns of genetic affinity of the TK-speaking group with modern and ancient Asian populations at different geographical scales were first inferred from the principal component analysis (PCA) (Fig. 2). We replicate the geography-related and language-related genetic patterns similar to those reported in previous studies [15, 22]. Our PCA plot reveals a clear separation between East Asian populations (on the right side of the PCA) and South Asian populations (on the left side of the PCA) along PC1. The Turkic-speaking population is positioned between these two regional groups. Meanwhile, along PC2, East Asian populations separate into two groups: Northeast Asian (top right of the PCA) and Southeast Asian (bottom right of the PCA), with Sinitic-speaking populations situated between these two regions.

To facilitate observation, we provide an additional PCA focusing on Southeast Asian populations. The results reveal partial clustering overlap between TK speakers from China and Southeast Asia, with our newly genotyped Lue and Yong samples positioned intermediate to these groups, along with some ancient samples from China and Taiwan, such as IA_Gongguan_TW, IA_Hanben_TW. Southeast Asian TK populations exhibit considerable genetic diversity, particularly along PC2, and show substantial genetic affinity with the AA and AN groups. In contrast, populations speaking Tibeto-Burman languages of the Sino-Tibetan (ST) family and Hmong-Mien (HM) languages are more genetically distinct from Southeast Asian TK speakers (Fig. 2).

**Fig. 3:**
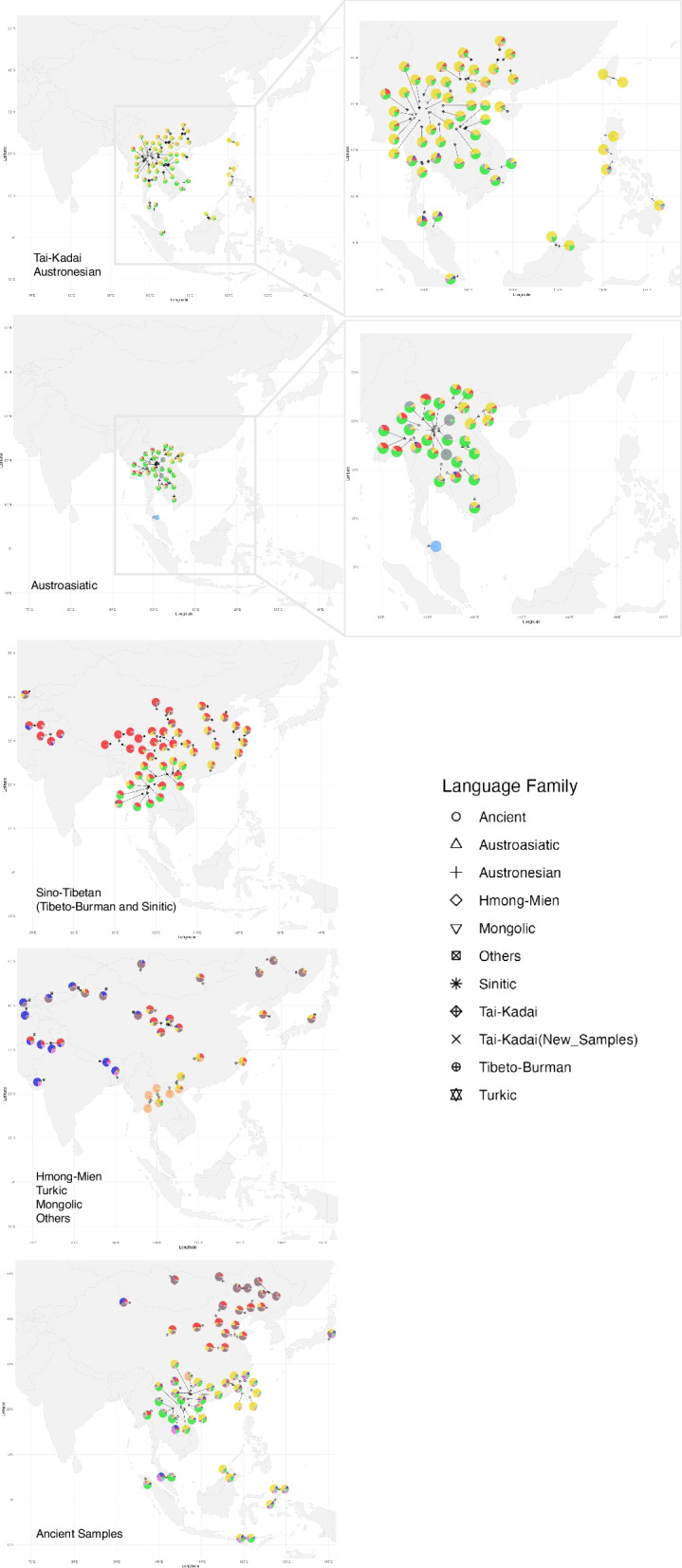
Geographic distribution of genetic composition inferred from ADMIXTURE at *K* = 10 in modern populations and ancient samples. Each population is represented by a symbol, with different shapes indicating language families, and colors representing the proportion of each ancestry component. Geographically distant outlier populations (e.g., French and Mbuti) were excluded to improve clarity and focus on the East Asian region. Population locations are plotted according to geographic coordinates, and the background map was created using *ggplot2* in R software

To dissect the ancestral components and genetic similarity of our studied group, we conducted a model-based ADMIXTURE analysis among 252 modern and ancient populations (Additional file 2: Fig. S1 and S2). When *K* = 10 (Fig. 3 and Additional file 2: Fig. S2), we observed four ancestries related to language family groups in East Asia: ST-related (red), AA-related (green), HM-related (orange), and TK/AN-related (yellow). Additionally, there are other components specific to some populations, such as the blue component in Indo-European and the pink component in the Onge population.

Focusing on the TK linguistic family (Fig. 3), these people exhibit a genetic structure composed of two main components (green and yellow), with varying proportions in TK populations in different regions. Other components, including the red component of ST, the orange component of HM, and the blue component of Indo-European, are mostly found in the TK population in central and southern Thailand. In the northern region of Thailand and southern China, TK-speaking populations, such as the Lue, Li, and Maonan, have a higher proportion of the yellow component compared to other TK-speaking populations. Even as *K* values increase, this genetic component remains prominent in these populations (Additional file 2: Fig. S3). The primary major ancestry components assigned to the TK-speaking population, as shown in yellow, are maximized in ancient samples from China and Taiwan (N_Penghu_CN, IA_Gongguan_TW, IA_Hanben_TW), while the second major component (green) is maximized in some Neolithic to Bronze Age samples from Southeast Asia, such as N.BA_TamPaLing_LA, BA_TamHang_LA, N_MaiDaDieu_VN, N_HhonHaiCoTien_VN, and N_GuaChaCave_MY (Fig. 3 and Additional file 2: Fig. S2).

To measure the pattern of genetic similarity for pairwise populations, we performed allele sharing-based outgroup-*f*_*3*_ analysis. The form of *f*_*3*_ (*X*, *Y*; outgroup) represents a statistical measure assessing the genetic affinity between populations* X* and *Y* when separated from the outgroup (Mbuti population). Higher values of this statistic indicate closer genetic relationships between populations. The outgroup-*f*_*3*_ demonstrates that populations speaking Indo-European, Dravidian, and Turkic languages, along with certain hunter-gatherer groups like the Maniq, Birhor, and Onge, exhibit distinct genetic differentiation from other East Asian populations, while the HM-speaking populations show the strongest sharing with each other (Additional file 1: Table S3 and Additional file 2: Fig. S4).

**Fig. 4:**
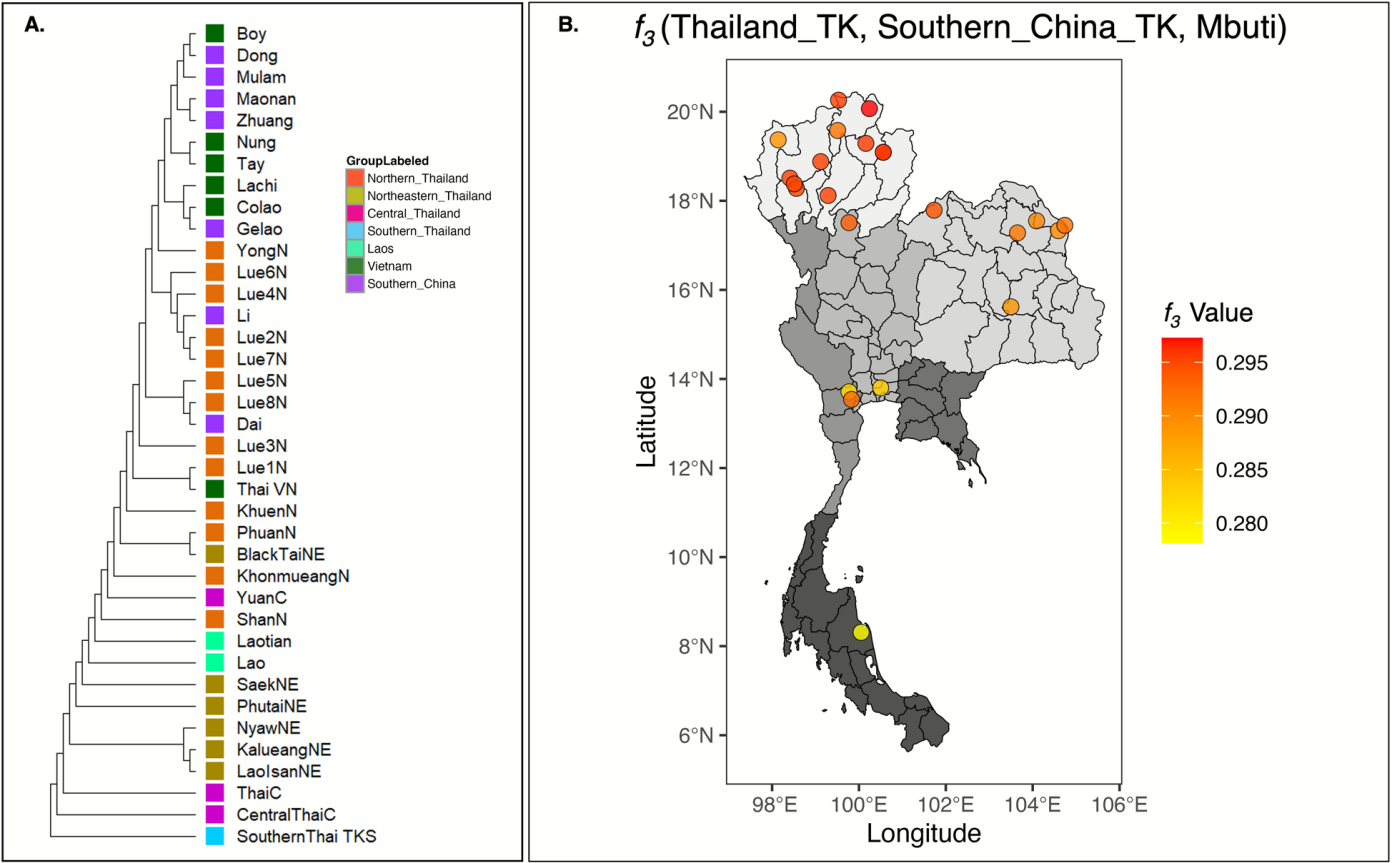
Genetic and geographic affinity among TK populations. **A** Neighbor-Joining tree constructed using genetic distances derived from pairwise 1 − *f*_*3*_ (Mbuti; *X*, *Y*) values among TK populations. **B** Spatial distribution of genetic affinity between TK populations in Thailand and southern China, visualized by color-scaled outgroup *f*_*3*_ values in the form *f₃* (Thailand_TK, Southern_China_TK; Mbuti). Population locations are plotted according to geographic coordinates, with warmer colors indicating higher allele sharing as indicated on the right panel. The background map was created using *ggplot2* in R software

Among the TK-speaking groups, outgroup *f₃* statistics in the form of *f₃*(TK, TK; Mbuti), along with a Neighbor-Joining (NJ) tree constructed from pairwise genetic distances (1 − outgroup *f₃*), indicate that TK populations in northern and northeastern Thailand exhibit high genetic affinity, clustering closely with ethnolinguistic groups from Vietnam and southern China (Fig. 4A and Additional file 2: Fig. S5). In contrast, TK populations from central and southern Thailand form separate branches, reflecting their more divergent genetic profiles. To further explore allele sharing between TK populations in Thailand and southern China, we modeled *f₃* (Thailand_TK, Southern_China_TK; Mbuti) using various TK-speaking groups in southern China as references, including Dong, Maonan, Dai, Zhuang, Gelao, Li, and Mulam. The resulting outgroup *f₃* values were consistently similar across all reference sources (Additional file 2: Fig. S6). A consensus spatial distribution map based on these results (Fig. 4B) supports the NJ tree topology, showing that TK populations from northern and northeastern Thailand share a higher level of allele affinity with TK groups in southern China. In contrast, the relatively reduced allele sharing in central and southern TK populations of Thailand suggests greater levels of genetic differentiation.

When comparing modern ethnolinguistic groups and ancient samples using outgroup-*f*_*3*_ (X, Ancient; Mbuti) (Additional file 1: Table S3 and Additional file 2: Fig. S7), we found a strong genetic connection between the TK populations in Thailand and ancient East Asian DNA samples, spanning from the Neolithic to the Iron Age, particularly across the Yellow and Mekong River basins. Notably, the TK populations in Thailand show higher outgroup-*f*_*3*_ values with ancient East Asian samples than with those from present-day Southeast Asia, such as IA_LongLongRak_TH and BA_TamHang_LA, suggesting a genetic continuity rooted in East Asia. This signal of ancient East Asian ancestry, indicated by high outgroup-*f*_*3*_ values, is more prominent among TK populations in northern Thailand compared to those in other regions.

**Fig. 5:**
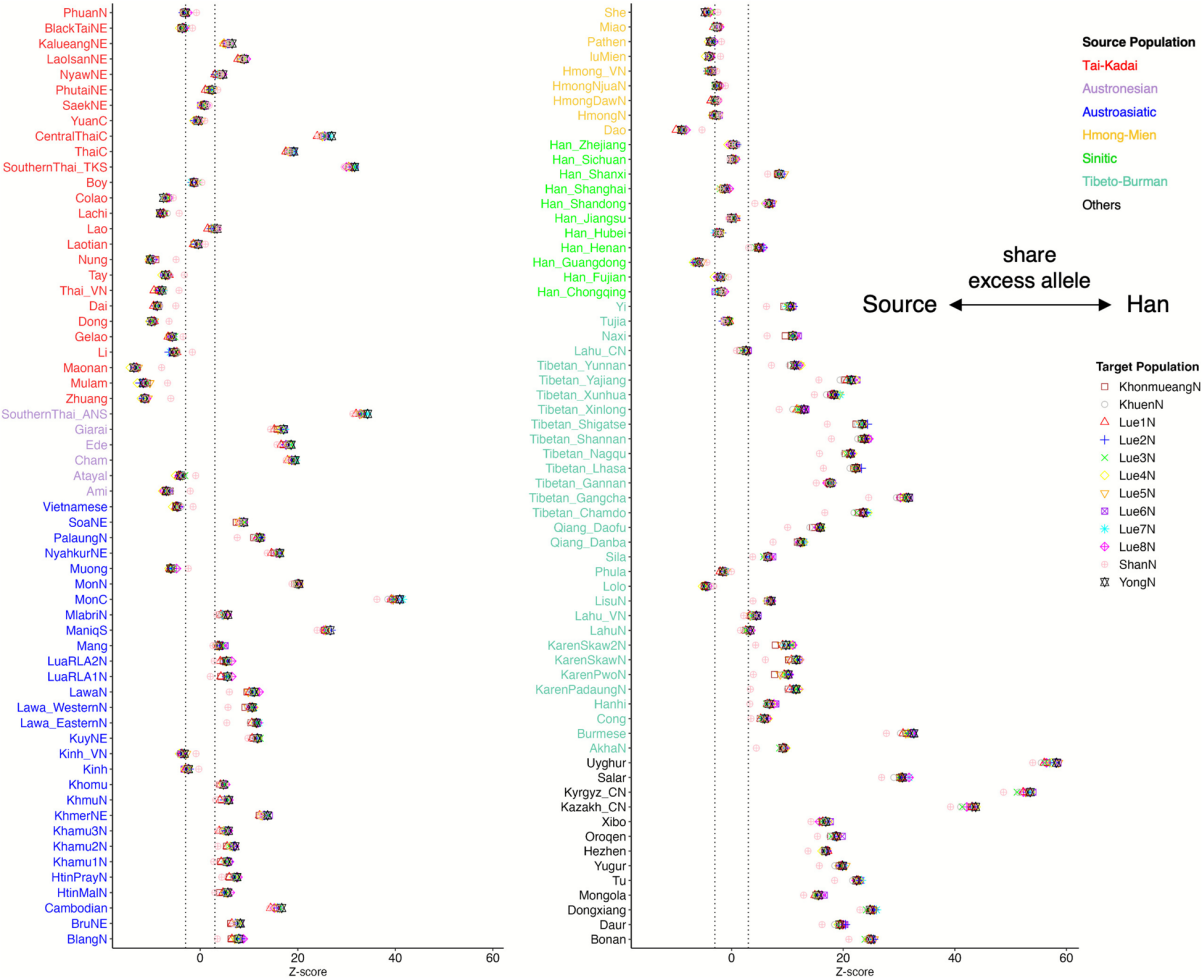
*f*_*4*_ statistics comparing TK speakers in northern Thailand from two different sources, Han Chinese and East Asians. *Z*-scores are for *f*_*4*_ (Han Chinese, East Asian; Target, Mbuti), where the target is the TK in northern Thailand. Different symbols denote different populations for the target. The colored label of the ethnic name is according to their language family. The vertical dashed lines denote + 3/− 3

**Fig. 6:**
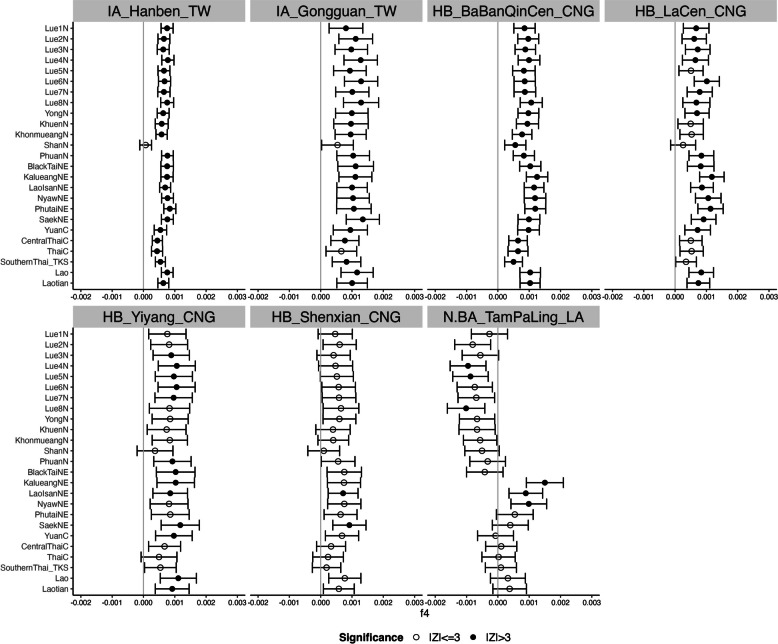
*f*_*4*_ statistics comparing TK-speaking ethnic groups in Thailand and neighboring countries to the ancient DNA from the Yellow River to Mekong regions. The figure shows only sites where at least one of the TK groups is significant. *Z*-scores are for *f*_*4*_ (Ancient sample, Han Chinese; Target, French), where the target is the Southeast Asian ethnic group. The vertical gray lines denote 0. Empty circles denote nonsignificant *Z*-scores (|*Z*|≤ 3), while solid circles denote significant *Z*-scores (|*Z*|> 3). Abbreviation: N Neolithic, IA Iron Age, BA Bronze Age, HB Historical/Burial caves, TW Taiwan, CNG China Guangxi, LA Laos

To investigate whether the TK populations in northern Thailand exhibit distinct affinities with East Asian sources, we computed *f*_*4*_-statistics in the form *f*_*4*_ (Han Chinese, East Asian; Target, Mbuti). A *Z*-score > 3 or < − 3 indicates a significant excess of ancestry shared with either Han Chinese or East Asians, respectively. Nonsignificant *Z*-scores suggest that Han Chinese and East Asians form a clade in relation to the target TK population in northern Thailand. Our analysis revealed that most TK in northern Thailand show a highly similar genetic profile, with strong affinities to other TK groups, especially those in southern China, such as the Maonan, Mulam, and Zhuang (Fig. 5 and Additional file 1: Table S4). However, the Shan population shows a slightly different pattern, exhibiting additional genetic affinities with some ST and AA-speaking groups, such as the Tibetan, Karen, and Lawa. To further examine affinities between modern populations and ancient samples, we employed *f*_*4*_-statistics in the form *f*_*4*_ (Ancient sample, Han Chinese; Target, French). The French population was used as an outgroup in this analysis instead of the Mbuti to reduce attraction artifacts associated with deep outgroups and to minimize potential noise from DNA damage patterns commonly observed in ancient DNA [23]. This approach allowed us to assess whether any targeted TK populations in Thailand or neighboring countries displayed closer affinity to ancient DNA from the Yellow River to Mekong regions compared to the Han Chinese. Except for the Shan, all TK-speaking groups in Thailand exhibit excess ancestry shared with certain Iron Age and Historical/Burial cave samples from Taiwan and China, e.g., IA_Hanben_TW and HB_BaBanQinCen_CNG (Fig. 6). Notably, there were no significant *Z*-scores between TK speakers in northern Thailand and ancient Southeast Asian DNA, such as N.BA_TamPaLing_LA and HB_TamHang_LA, which are typically linked to AA-speaking populations (Additional file 1: Table S5 and Additional file 2: Fig. S8).

**Fig. 7:**
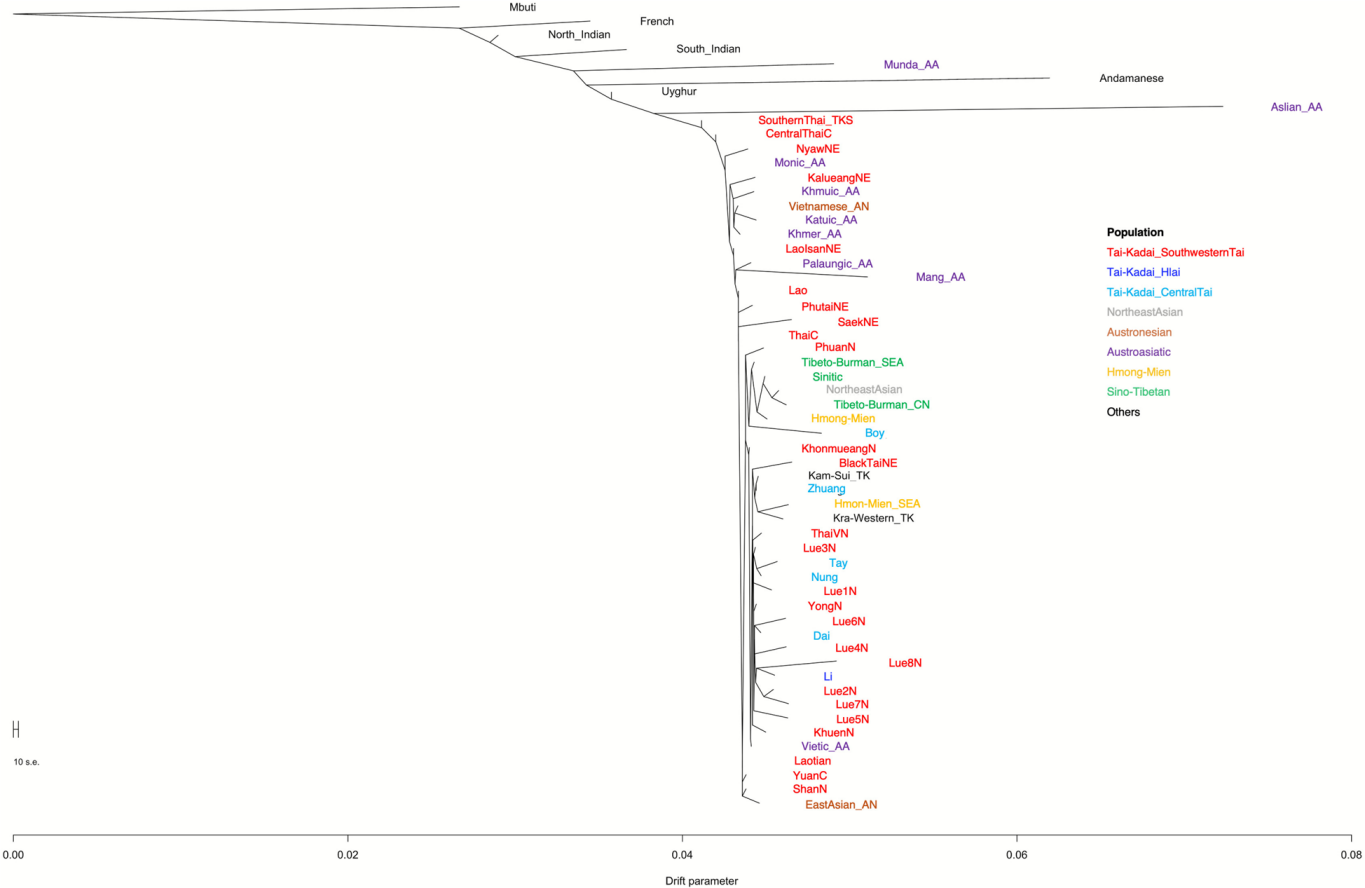
TreeMix diagram without migration events for the TK-speaking ethnic groups in Thailand (red) and other modern populations in East and Southeast Asia. Reference populations are labeled with different colors based on their language family. The TK populations are separated by ethnicity, while other ethnolinguistic groups are grouped according to their language family

We constructed a maximum-likelihood tree using genome-wide data from TK speakers in Thailand and neighboring countries, along with diverse East Asian populations, utilizing the TreeMix program version 1.12. Our findings indicate that TK groups in northern Thailand, such as the Lue, Yong, and Khuen, cluster closely with various TK ethnolinguistic branches predominantly found in southern China and Vietnam. In contrast, TK speakers in northeastern Thailand, including the Kalueang and Lao Isan, show closer genetic affinity with indigenous AA-speaking groups in Thailand, suggesting possible historical admixture and prolonged interactions with local AA groups (Fig. 7). When accounting for migration events in the TreeMix analysis, we observe an additional genetic link between Indian lineages and a clade of TK populations in northern Thailand (Additional file 2: Fig. S9).

### Haplotype-based analyses for genetic admixture and ancestry

**Fig. 8:**
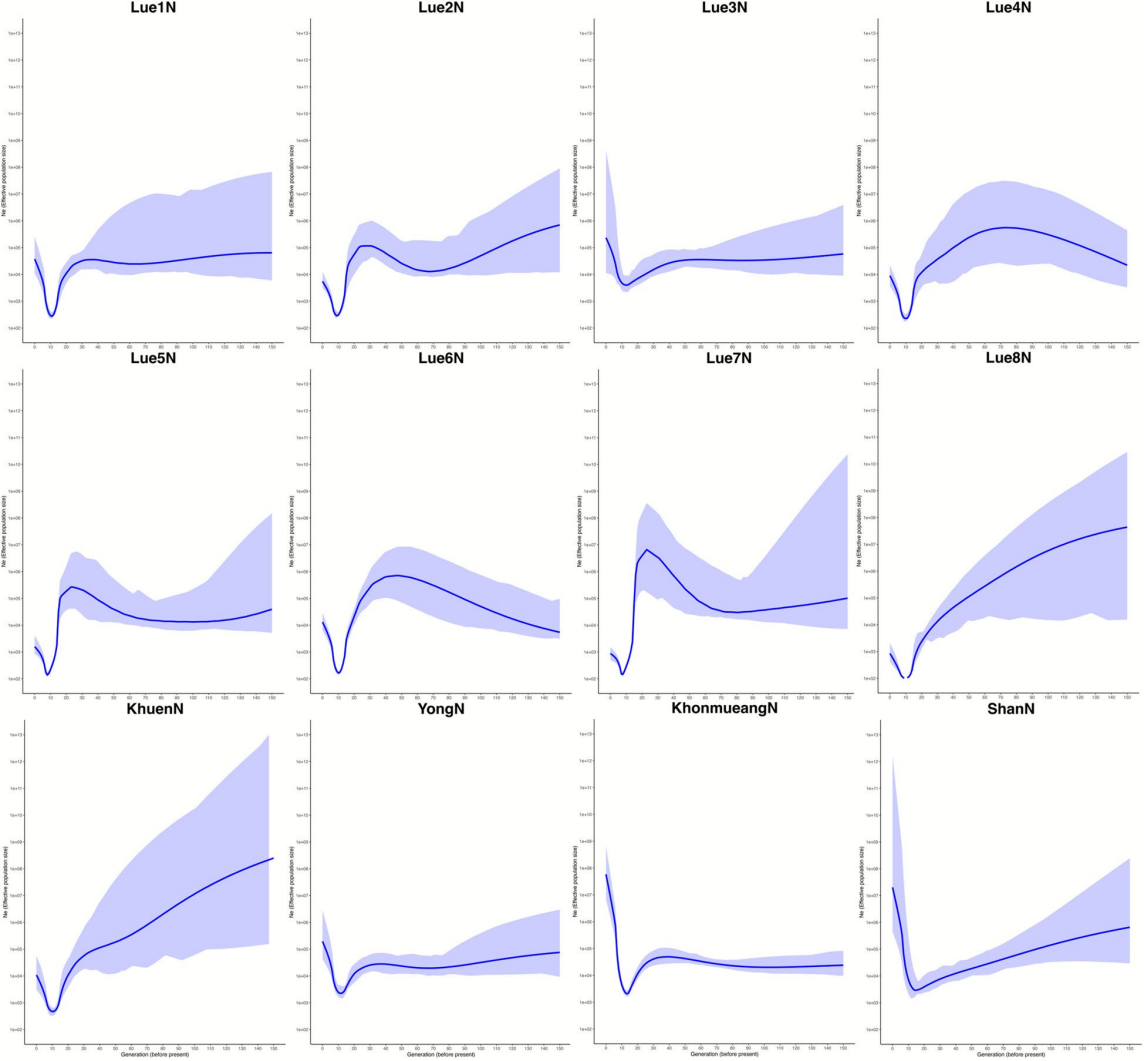
Estimates of effective population sizes for each TK-speaking ethnic groups in northern Thailand over the past 150 generations

All allele-based descriptive analyses indicate the genetic continuity of the TK populations across the upper GMS, especially from southern China to Thailand. In Thailand, the concentration of the TK gene pool seems to be in a north-to-south direction, with TK in the northern region showing a closer genetic relationship with TK in southern China than those in northeastern, central, and southern Thailand. To further analyze the details of the ancestry and admixture composition of TK in Thailand, we employed haplotype-based analysis. The haplotype-sharing profiles, as inferred by IBD analysis, also reinforce that TK speakers in Thailand and southern China are very genetically similar. Some TK-speaking groups in Thailand, such as Lue2N from Nan province and Lue7N from Phayao province, exhibit a high degree of IBD sharing, reflecting their shared genetic history (Additional file 2: Fig. S10). Focusing on the genetic history of TK in northern Thailand, we estimated the effective population size over the last 150 generations. We found that all of the TK ethnic groups in northern Thailand have experienced rapid declines in population size during the past 10 generations, corresponding to the period around 250–300 years ago. Overall, the decline in population size has been stronger in the Lue and Khuen ethnicities compared to the Yong, KhonMueang, and Shan (Fig. 8). While TK speakers in southern China and northern Vietnam, such as the Boy ethnic group, exhibit slight signals of a population size decrease, others, including the Zhuang and Maonan, have experienced gradual increases in population size, reaching their current maximum (Additional file 2: Fig. S11).

**Fig. 9:**
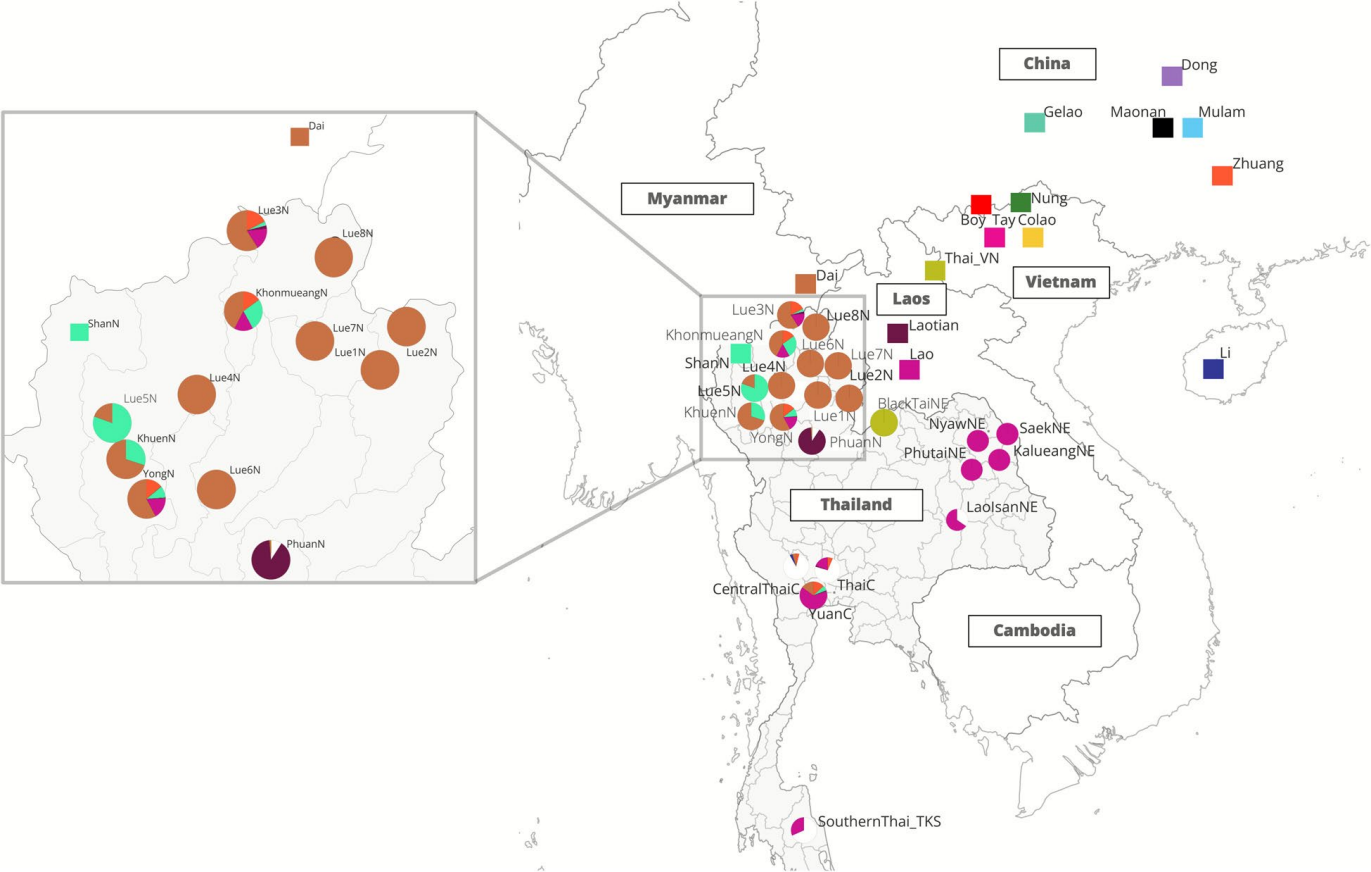
Map showing the admixture proportions in the TK populations in Thailand (circles), estimated using SOURCEFIND. The TK surrogates residing outside Thailand are represented by square boxes in different colors. Ancestry from other language groups is displayed in white. The background map was created using QGIS 3.6.0 (http://www.qgis.org/)

**Fig. 10:**
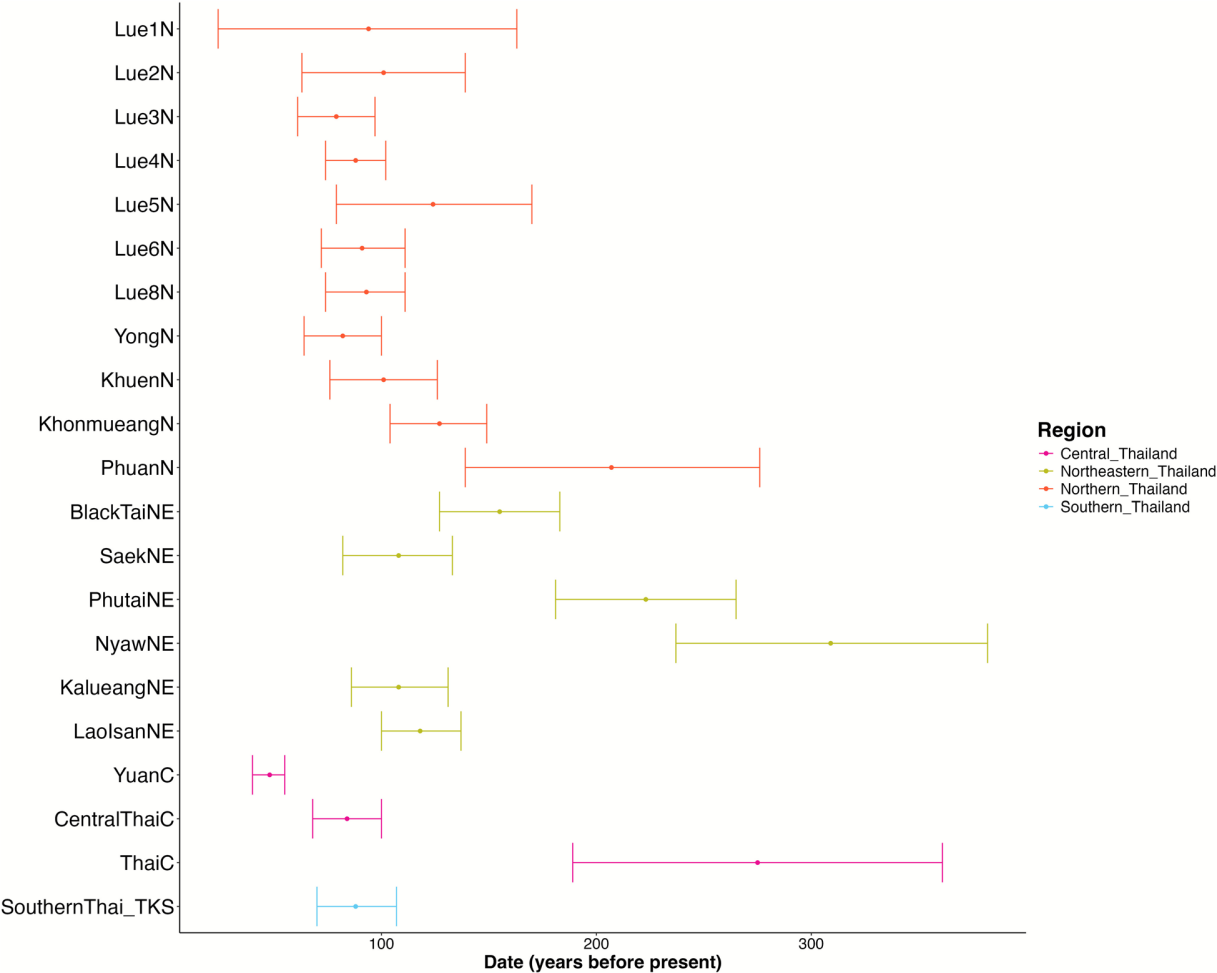
Admixture time estimated by fastGLOBETROTTER in the TK populations in Thailand. The error bars denote the 95% confidence interval

We next used the SOURCEFIND, based on the output of ChromoPainter, to identify proxies for the admixture sources in the TK populations of Thailand. We included various ethnolinguistic groups residing in East and Southeast Asia in the source estimation (Additional file 1: Table S6). The TK-speaking Shan ethnic group was also selected as one of the surrogates, even though they live in northern Thailand, due to their genetic differentiation and recent migration history. The program assessed the coancestry matrix to infer the proportions of genetic contribution from donor populations into each target population. We specifically focused on the TK-speaking surrogates, and the map of their genetic contributions to the TK populations in Thailand is presented in Fig. 9. We found that the TK populations in Thailand had genetic proportions predominantly from Dai-, Zhuang-, Shan-, and Lao-related ancestries (Additional file 2: Fig. S12). It is crucial to recognize that the term “Dai-related” used in our admixture analysis refers not to the present-day Dai population per se, but to a surrogate ancestral gene pool that shares close genetic affinity with the Dai. This terminology is also used with other ethnic-related to broadly represent ancestral sources in this admixture inference process. The Dai-related ancestry is the dominant major source in the TK populations of northern Thailand, with their proportions varying across populations, ranging from 19.6 to 100% (Additional file 1: Table S7). For the TK populations in other regions, the dominant proxy for the major source is the Lao-related ancestry, ranging from 19% in ThaiC in the central region to 100% in some TK-speaking groups in northeastern Thailand, such as NyawNE and PhutaiNE. Interestingly, some TK populations, such as ThaiC, Central_Thai, and Southern_Thai, exhibited the major source from other ethnolinguistic groups, with the TK-related ancestry as a minor source (Fig. 9 and Additional file 2: Fig. S12). We then used fastGLOBETROTTER program to estimate the timing of admixture events using shared haplotype size distribution. The admixture dates in TK speakers are very recent, between 100 and 300 years ago, with the NyawNE tending to be older than those of the other TK populations in Thailand (Fig. 10 and Additional file 1: Table S8). Notably, the recent admixture dates inferred for TK populations in Thailand reflect gene flow from the most probable surrogate sources, which predominantly appear to be TK-related groups from southern China and Laos. These dominant sources may suppress signals from older ancestry components that persist at lower proportions.

### Shared selection

We detected SNP positions showing signals of positive selection in our newly generated dataset using the population branch statistics (PBS). In the Lue population, we identified 325 SNPs within the top 0.1% PBS outliers. After excluding signals likely associated with ancient selection, 312 positions remained. The SNPs with the highest PBS value (2.484) was rs73213504, located in the *FER* (FPS/FES-related tyrosine kinase) gene on chromosome 5, which is involved in intracellular signaling, cell adhesion, and angiogenesis (Additional file 1: Table S9).

Among the positively selected SNPs in the Lue, 83 positions annotated to 38 genes were shared with TK populations from southern China. The Lue population shared the greatest overlap with the Gelao ethnic group (40 SNPs) and the least with the Dai and Dong populations (9 SNPs each) (Additional file 2: Fig. S13). Two SNP positions, rs2274379 and rs12426969, were frequently shared across TK-speaking populations, except for the Zhuang and Mulam. The rs2274379 SNP is located in the *DOCK1* gene on chromosome 10, which encodes a protein involved in cytoskeletal dynamics and neuronal development, while rs12426969 is in the *LRIG3* gene on chromosome 12, a regulator of receptor tyrosine kinase signaling associated with tissue morphogenesis and cell growth.

We also observed cases in which, although the positively selected SNP positions differed among TK populations, several of these SNPs were located within the same gene. For example, *VEPH1* on chromosome 3, known to regulate developmental signaling pathways, was under positive selection in the Lue population at multiple SNPs (rs73156789, rs73156774, rs6795781, and rs16827507), whereas in the Dai and Dong populations, a different SNP within the same gene (rs35193163) showed evidence of selection (Additional file 1: Table S9). This pattern suggests that the gene itself may be under selection pressure across TK-speaking populations.

## Discussion

The genetic structure within ethnic groups in the upper GMS, stretching from southern China to Thailand, shows a history of migration and settlement by various communities dating back to ancient times, as shown in both archeology and history. The genetic evidence in this study provides insight into the genetic structure and relationships between populations in East and Southeast Asia, which differ from ethnic groups in other parts of the world, such as Northern Asia and the Indian subcontinent (Fig. 2). However, it is important to note that populations in this region still show high genetic diversity. Each language family, TK, AA, ST, HM, and AN, has its own unique genetic structure. The migration and interaction among people in southern China and Southeast Asia since prehistorical and historical times have contributed to the genetic and cultural diversity in this area. Over hundreds of years, TK-speaking people migrated south from their homeland in southern China and became the majority population in Thailand and Laos.

Among the different ethnolinguistic groups to which modern Southeast Asian populations belong, the genetic components analyzed by ADMIXTURE show a close relationship between the TK and AN populations, as both share a similar ancestry pattern mainly made up of green and yellow components (Fig. 3). The TK and AN-speaking populations share a complex historical and linguistic relationship, suggesting a deep connection [24]. Linguistic evidence indicates that the TK language family may have split from the AN language family, with some scholars proposing a shared origin in the coastal regions of southeastern China or Taiwan [25]. Genetic studies also show overlapping ancestry, reflecting ancient interactions and migrations between these groups [15, 26, 27]. TK speakers likely migrated south into mainland Southeast Asia, while AN speakers expanded across island Southeast Asia and the Pacific [11]. The similar genetic composition found in our results supports the shared ancestry and historical interaction between people from these two language families.

Comparing modern and ancient DNA has provided insight into the origin of the TK people of Thailand, as shown by the green and yellow components in Fig. 3. The yellow component is likely inherited from East Asian ancestry, supported by its presence in ancient DNA samples from East Asia, such as China and Taiwan. The green component, which peaks in some Neolithic to Bronze Age samples from Southeast Asia, is likely linked to present-day AA speakers. The genetic composition of TK populations in Thailand shows a strong East Asian-related ancestry, followed by Southeast Asian-related components, with some contributions from other language groups. This suggests that TK-speaking populations in Thailand inherited their East Asian ancestry through migrations from southern China, then mixed with indigenous AA groups, who have lived in the area since at least the Neolithic period. The presence of the yellow component across these populations supports the historical migration across the upper GMS, shaping the genetic ancestry of TK populations in Thailand. It is noteworthy that in addition to the two major ancestral components, TK speakers in Thailand also exhibit minor genetic contributions from other linguistically related groups, such as ST and HM. These components may have been introduced through historical interactions between TK populations and their neighboring groups. For instance, historical records indicate substantial Han Chinese migration into Yunnan, one of the proposed homeland of TK ancestors, during the Ming Dynasty (1368–1644 AD) [28]. Furthermore, some scholars have proposed that some present-day Han Chinese populations may be partially descended from local TK-speaking groups that experienced language shifts toward Chinese dialects [29]. Therefore, the presence of ST-related genetic components in TK-speaking populations may reflect both deep shared ancestry and more recent admixture events.

Estimates of ancestral proportions using SOURCEFIND have revealed regional differentiation in Thailand. TK populations in northern and northeastern Thailand show a higher proportion of TK-related ancestry compared to those in central and southern regions, where genetic contributions from non-TK speaking ancestry exceed 50% (Fig. 9). TK speakers in Thailand mostly have ancestry from at least one of the TK-speaking Dai-, Zhuang-, Shan-, or Lao-related ancestries, except for the BlackThaiNE which shows a major Thai_VN-related component. Interestingly, the CentralThaiC, ThaiC, and SouthernThai_TKS contain ancestry related to the Indian subcontinent, such as Bengali- and Gujarati-related components (Additional file 2: Table S7). This is consistent with previous reports on the Indian heritage of the people of Thailand. Maritime trade networks linking the Indian subcontinent with mainland Southeast Asia likely facilitated this admixture, especially in central and southern Thailand [15, 26, 30].

Focusing on the TK-related component, the ancestral lineage of the TK people in northern Thailand comes from Dai-related ancestry, while the other part comes from Lao-related ancestry. The proportion of TK gene pool from southern China seems to follow a north-to-south gradient, with TK populations in northern Thailand showing closer genetic ties to those in southern China than to their counterparts in northeastern, central, or southern Thailand (Fig. 9). The history of TK migration from southern China to northern Thailand became well known by the late first millennium CE, driven by factors such as population density, the need for agricultural land, and political upheavals in China [19]. The TK groups gradually moved southward along river systems in the upper GMS, establishing settlements and interacting with indigenous AA populations. Their arrival in northern Thailand led to the formation of early polities, such as the Lan Na Kingdom, and the integration of their language and culture into the region [18]. Although the historical migration of the TK people may have occurred for at least the past thousand years, the influx of TK people from southern China into northern Thailand likely intensified in the most recent period. Our estimation of the admixture date for the TK-related ancestry is between 100 and 300 years ago (Fig. 10), which is consistent with historical evidence of the large-scale TK migration. The most well-known migration of TK people into northern Thailand is linked to historical events, especially during the late eighteenth and early nineteenth centuries under King Kawila of the Lan Na Kingdom [10]. As part of his resettlement campaigns, famously referred to as “gather vegetables into baskets, gather people into cities,” King Kawila sought to rebuild and repopulate the war-torn cities of the Lan Na Kingdom, in what is now northern Thailand [31, 32]. This campaign involved relocating entire communities, including various TK-speaking groups like the Lue, Yong, and Khuen people, from areas such as the Xishuangbanna region of southern China and the Shan State of Myanmar, to revitalize the depopulated northern Thailand region. Many TK people were resettled in key areas like Chiang Mai, Lampang, and Lamphun provinces. This resettlement not only strengthened the economic and cultural vitality of the Lan Na Kingdom but also preserved the TK heritage from southern China, which continues to influence northern Thailand today [32].

Among the TK populations in northern Thailand, the Lue exhibit a majority of their ancestral components derived from Dai-related ancestries from southern China. However, the genetic composition varies among the Lue subgroups (Fig. 9). Previous reports using uniparental markers indicate that the genetic structure variations among Lue populations may result from the founder effect [33, 34]. This occurs when the Lue people migrated southward along the Mekong River and into northern Thailand via different migration routes, including those through the Thai/Laos or Thai/Myanmar borders. The migration patterns of the Lue people in each village were complex, as they adapted to different circumstances over different periods. The Lue resettlement scenario led to the impact of founder effects, which reduced genetic diversity, as seen in the widespread decline in effective population sizes approximately 250–300 years ago (Fig. 8), consistent with the timing of the Lue migration. Despite historical records indicating common origins and shared ancestry among the Lue communities in northern Thailand, migration from southern China to Thailand during the eighteenth century, both voluntary and involuntary, led to the fragmentation of the Lue into smaller groups. Each group followed different migration routes, timelines, and patterns, resulting in different genetic structures among Lue populations in northern Thailand today.

Interestingly, the YuanC population showed a different genetic composition compared to those living nearby in Central Thailand (Fig. 9). They exhibited a high proportion of TK-related ancestry and contained some specific components, such as Shan-related ancestry, which are mostly found in populations residing in the western part of northern Thailand. The Yuan people are recognized as the earliest TK speakers to settle in northern Thailand. By the eighth century A.D., they founded the first Tai kingdom, known as “Yonok” or “Chiang Saen,” in what is now Chiang Rai province [11]. In the early 1800 s, following the Siamese-Burmese war, the policy of the King of Siam (Thailand) led to the relocation of the Yuan people in Chiang Saen. As a result, they moved to central Thailand and resettled in provinces such as Uttaradit, Saraburi, and Ratchaburi [35]. Our results show that, even though the Yuan left their historical hometown in northern Thailand hundreds of years ago, they still preserve an ancestral composition similar to the TK people in northern Thailand. This is consistent with the fact that they preserve many northern Thai cultural features and speak a local language from the Tai family. These results shed light on the transmission of the TK genetic pathway from southern China, through northern Thailand, to the lower part of Mainland Southeast Asia.

Overall, this study, which used large-scale genomic data, reveals the origins and genetic proportions of various ethnic groups in Thailand. We demonstrated the continuity of the TK genetic lineages from southern China to Thailand, especially concentrated in the northern part. These findings provide important insights into the impact of migration on ethnolinguistic diversity and the spread of genetic disorders. For example, the varying prevalence and patterns of G6PD deficiency, such as the Kaiping (1388G > A) and Canton (1376G > T) types, were found in the northern Thai population, compared to other regions where the Viangchan (871 G > A) and Mahidol (487 G > A) types are more prevalent [36]. We also found that several SNPs under positive selection in our newly generated TK samples were shared with TK-speaking populations in southern China. This suggests common adaptive pressures driven by the environments, diets, or sociocultural contexts of their shared ancestors. However, it is important to note that our analysis is primarily based on genome-wide Human Origin array data, in which most of the SNP positions are in non-coding regions. As a result, the number of pathogenic variants detected is relatively low. A more comprehensive understanding of selection would require whole-genome sequencing data, which can capture a broader range of selection signals. Moreover, our analysis assumes that the populations used as the reference for the genetic makeup have unique and distinct genetic structures compared to other groups. In reality, these ethnic groups may be mixed populations or share some genetic characteristics with other communities, leading to potential inaccuracies in measuring genetic proportions. Increasing the diversity of reference populations could improve the accuracy in identifying genetic lineage sources.

## Conclusions

The genetic structure of ethnic groups in the upper GMS reflects a long history of migration and cultural exchange. Our genome-wide analyses show that the genetic composition of TK speakers in Thailand mainly reflects East Asian ancestry, with some Southeast Asian contributions, suggesting long-term migration from southern China to Thailand. Regional differences in Thailand show that northern TK speakers are genetically closer to people in southern China, while those in central and southern Thailand have more non-TK contributions. Among northern Thai populations, the highest genetic contributions come from Dai-related communities, while northeastern Thai speakers have the highest proportion of Lao-related ancestry. The genetic history of the TK-speaking Lue and Yuan populations further illustrates how migration patterns, founder effects, and historical resettlements have shaped genetic diversity. Overall, our study confirms the historical continuity of TK migration from southern China, shedding light on the genetic and cultural changes that have shaped modern populations in Thailand.

## Methods

### DNA extraction and quality control

White cell lysates obtained from 97 participants belonging to 6 TK-speaking Lue and 1 Yong populations (Fig. 1 and Additional file 1: Table S1), selected based on appropriate inclusion and exclusion criteria for population genetic studies [33, 36], were used for DNA extraction. Volunteers were healthy subjects who were over 20 years old, of Lue- or Yong-speaking ethnicity, and had no ancestors who were known to be from other recognized ethnic groups for at least three generations. We collected personal data using form-based oral interviews for unrelated lineages, ethnicity, and migration histories.

Subsequently, a total of 97 qualified samples were sent for genotyping using the Affymetrix Axiom Genome-Wide Human Origins array [21] at ATLAS Biolabs in Germany. A total of 629,721 SNPs per individual were obtained when compared to the human reference genome coordinates version hg19, with a genotype call rate of at least 97%. We used PLINK version 1.90b5.2 [37] to remove individuals with more than 5% missing data, as well as to exclude mitochondrial DNA and sex chromosomes from our analysis. Loci that did not pass the Hardy–Weinberg equilibrium test (*p*-value < 0.00005), had minor allele frequency (MAF) less than 3.3 × 10^−4^, or had more than 5% missing data within any population were also excluded. Additionally, we used KING 2.3 [38] to assess individual relationships and removed one person from each pair of first-degree kinship. After filtering, a total of 92 individuals with 618,511 SNPs were retained for further analysis (Additional file 1: Table S1).

We utilized the “bmerge” function in PLINK version 1.90b5.2 to combine our newly genotyped data with genome-wide SNP data from modern and ancient populations in East and Southeast Asia, as well as reference populations like the Mbuti and French from the Allen Ancient DNA Resource (AADR) version 54.1 [39]. Additionally, we incorporated data obtained from previous studies [6, 15, 20, 23, 26, 40]. For the modern samples, we assessed the data quality using PLINK version 1.90b5.2 and excluded SNPs with more than 5% missing data, had MAF less than 3.3 × 10^−4^, or were not in Hardy–Weinberg equilibrium at significance level of *p*-value < 0.00005. Meanwhile, the ancient samples with less than 15,000 SNP positions were excluded. This resulted in a dataset comprising 2284 individuals from 252 populations (Additional file 1: Table S2) with 501,356 SNP positions available for further analysis.

### Population structure and relationship analyses

We used the “indep-pairwise” command in PLINK version 1.90b5.2 to prune SNPs of the same linkage disequilibrium with an *r*^2^ value greater than 0.4 within windows of 200 variants and a step size of 25 variants. After excluding the Mbuti and French populations used as outgroups, there were 219,895 SNP positions available for analysis from a total of 2234 individuals. Principal component analysis (PCA) was carried out using *smartpca*, a part of the EIGENSOFT package [41]. We used all default parameters along with additional parameters of lsqproject: YES and autoshrink: YES. The ancient DNA data, which differs genetically from modern populations, was projected.

The ADMIXTURE, a model-based clustering algorithm for ancestry estimation method, was utilized to investigate the genetic composition of a merged dataset comprising 2284 individuals from 186 modern and 66 ancient populations (Additional file 1: Table S2). This analysis aimed to discern genetic structures and infer ancestral origins with respect to ethnicities and linguistic groups. We employed ADMIXTURE version 1.3.0 [42], varying the number of assumed ancestral components (*K*) from 2 to 13, and conducted 100 bootstrap iterations with 10 independently seeded runs per K. The results for each K were summarized across 10 replicates using PONG [43], with replicates having an average pairwise similarity threshold > 0.9 treated as supporting the same representative model. The ancestral components corresponding to the *K* value with the lowest cross-validation error were visualized using AncestryPainter [44] and R software [45].

We assessed shared genetic drift and signals of excess ancestry among the studied populations by calculating outgroup *f₃* and *f₄* statistics using ADMIXTOOLS version 5.1 [21], in combination with the admixr package version 0.7.1 [46]. The outgroup *f₃* matrix was visualized as a heatmap using the *pheatmap* package in R version 4.3.2. For the focal TK-speaking populations, outgroup *f₃* values were also plotted geographically with *ggplot2*, and subsequently converted into genetic distances (1 – outgroup *f₃*) to construct a NJ tree using the MEGA X software [47]. All *f₄*-statistic results were visualized using *ggplot2* in R software.

### TreeMix analysis

We constructed a TreeMix-based phylogenetic tree to infer genetic relationships and evaluate gene flow events among the TK-speaking and other East Asian populations, using the Mbuti, European French, and Indian populations as outgroups. Utilizing TreeMix version 1.13 [48], we reconstructed a phylogenetic tree with migration events ranging from 0 to 5. The TK population was separated by their ethnicity, while the comparing AA, ST, AN, and HM populations were grouped according to their language family (Additional file 1: Table S2).

### IBD estimation and effective population size

We utilized refined-ibd.17Jan20.102.jar [49] to estimate the shared IBD segments within and between TK individuals. We specified 1 cm as the minimum length for reported IBD segments (length = 0.1). We used the default values of other parameter settings to estimate individual pairwise. Subsequently, we calculated the average total IBD among populations based on the length of individual IBD segments, distinguishing between those ranging from 1–5 to > 5 cm, reflecting ancient genetic interactions occurring between 500 and 1500 years ago and those within the last 500 years, respectively [23]. The ibdne.23Apr20.ae9.jar [50] was used to estimate the effective population size among geographically diverse TK speaking populations in northern Thailand and southern China over the last 150 generations.

### Admixture source and date inference

We phased 185 modern populations using SHAPEIT v2 software [51] with the HapMap phase II b37 [52]. We then ran ChomoPainter v2 [53] on the phased dataset to investigate haplotype sharing. We first estimated switch rate and global mutation probability by running ChomoPainter v2 on chromosome 1, 5, 10, 15, and 20 using 10 expectation maximization (EM) iterations. We randomly down-sampled each population to 2 samples for this EM process with “-a” argument to identify the chromosome assignments for each individual, with all modern populations serving as both donors and recipients. The switch rate and mutation probability from the EM estimation were then used as the starting values for these parameters for all individuals of donors and recipients in the painting process.

To figure out the ancestral haplotype compositions and surrogates of TK speakers in Thailand, we employed SOURCEFIND [54] using the following setting: num.surrogates, 8; exp.num.surrogates, 4; num.iterations, 200,000; num.burnin, 50,000; and num.thin, 5000. In this analysis, we designated all 22 TK populations in Thailand as the target, with 128 world-wide populations serving as surrogates (Additional file 1: Table S6). Admixture proportions were averaged across 30 posterior samples, and ancestry components contributing less than 1% were excluded from further interpretation. We further applied fastGLOBETROTTER [55] on the same set of target and surrogate for inferring the best-fitting admixture models and estimating admixture dates with default parameters, using bootstrap.num 100 and num.admixdates.bootstrap 2. When conclusions were deemed “uncertain,” the result from “1-DATE FIT SOURCE, PC1” was used to represent the basic admixture model. Admixture dates were calculated with a 95% confidence interval derived from 100 bootstrap iterations.

### Selection analysis

We used a modified version of the population branch statistics (PBS) method [56] to detect population- and region-specific signals of natural selection. PBS was calculated using the following formula: PBS_A_ = (T_AB_ + T_AC_ − T_BC_)/2, where T = − log(1 − F_ST_), and A is the target population, B is the ingroup, and C is the outgroup.

In the first comparison, the Lue population was set as the target (A), Han_Shanxi as the ingroup (B), and the French from the HGDP dataset as the outgroup (C), to identify ancient selection signals. In the second comparison, Lue was again used as the target population, with Li as the ingroup and Han_Shanxi as the outgroup, aiming to detect region-specific selection.

Variants in the top 0.1% of PBS values were considered candidate loci under selection. To isolate region-specific signals from broader ancient selection, we filtered out overlapping SNPs between the two comparisons using the *setdiff()* function in R. Only loci unique to the regional comparison were retained for downstream analysis.

This two-comparison framework was also applied to other TK-speaking populations from southern China (Dai, Zhuang, Dong, Gelao, Mulam, and Maonan), each treated as the target group. After identifying candidate SNPs, shared loci between Lue and each TK population in southern China were determined using the *intersect()* function in R. All SNPs were annotated using the Axiom Human Origins Array annotation file (https://www.thermofisher.com/th/en/home/life-science/microarray-analysis/microarray-data-analysis/genechip-array-annotation-files.html). Finally, PBS results were visualized as Manhattan plots using *ggplot2* in R, with top 0.1% threshold marked and shared candidate genes labeled.

## Supplementary Information

Additional file 1: Table S1 General information on samples whose data were newly generated in this study. Table S2 Data of populations using for analyzed dataset. Table S3 Result of the *f*_*3*_ (X, Y: Outgroup), where Outgroup = Mbuti. Table S4 Result of the *f*_*4*_ (W, X: Y, Outgroup), where Outgroup = Mbuti. Table S5 Result of the *f*_*4*_ (W, X: Y, Outgroup), where Outgroup = French, W are Ancient groups. Table S6 Surrogates and Targets population for SOURCEFIND and fastGLOBETROTTER analysis. Table S7 Results of SOURCEFIND analysis. Table S8 Results of fastGLOBETROTTER analysis. Table S9 The table lists SNP positions and related information within the top 0.1% PBS scores from the analysis using the Lue population as the target, Li as the ingroup, and Han_Shanxi as the outgroup.

Additional file 2: Fig. S1 The mean cross-validation values of 10 run ADMIXTURE ranging from K = 2 to 13. Fig. S2 Unsupervised ADMIXTURE diagram illustrates the genetic components of modern and ancient populations in South Asia, Northeast Asia, and Southeast Asia, delineated into K = 10 groups of ancestral components with AncestryPainter. Each individual is depicted by a bar segmented into K colored sections, representing their estimated ancestry components. Populations are demarcated by black lines, and their linguistic family is labeled outside the diagram. Newly generated samples are labeled in red by their population names. [Abbreviation for ancient samples: Ho = Hoabinhian, P = Paleolithic, M = Mesolithic, N = Neolithic, IA = Iron Age, BA = Bronze Age, HB = Historical/Burial caves, TW = Taiwan, CN = China, CNG = China Guangxi, LA = Laos, TH = Thailand, VN = Vietnam, ID = Indonesia, MY = Malaysia, MN = Mongolia, RU = Russia, SA = South Africa, JP = Japan, US = United States; Suffix abbreviation for TK samples in Thailand: N = Northern, NE = Northeastern, C = Central, and S = Southern]. Fig. S3 ADMIXTURE diagram showing the genetic components of various East and South Asian populations. K values ranging from 2 to 13 divided into groups from K = 2 to K = 13 using PONG program. Each individual is represented by a bar divided into K colored segments, indicating their estimated membership fractions in each of the K ancestry component. Fig. S4 Quantitative measurement for pairwise genetic affinity based on allele sharing. Outgroup-*f*_*3*_ in the form *f*_*3*_(Mbuti; X, Y) measuring shared genetic drift between pairwise modern populations. Fig. S5 Quantitative measurement of genetic affinity between pairwise TK-speaking populations based on outgroup-*f*_*3*_ in the form *f*_*3*_(X, Y; Mbuti). Fig. S6 Geographic distribution of genetic affinity between TK populations in Thailand and Southern China, visualized by color-scaled outgroup *f*_*3*_ values in the form *f*_*3*_(Thailand_TK, Southern_China_TK; Mbuti). Population locations are plotted according to geographic coordinates, with warmer colors indicating higher allele sharing. Fig. S7 Quantitative measurement for pairwise genetic affinity based on allele sharing. Outgroup-*f*_*3*_ in the form *f*_*3*_(Mbuti; X, Y) measuring shared genetic drift between pairwise modern and ancient populations. Fig. S8 *f*_*4*_ statistics comparing TK-speaking ethnic groups in Thailand and neighboring countries to the ancient DNA from the Yellow River to Mekong regions. Z-scores are for *f4* (Ancient sample, Han Chinese; Target, French), where the target is the Southeast Asian ethnic group. The vertical gray lines denote 0. Ethnic names are colored according to language family. Empty circles denote nonsignificant Z-scores (|Z|≤ 3), while solid circles denote significant Z-scores (|Z|> 3). Fig. S9 TreeMix diagram with 1–5 migration events for the TK-speaking ethnic groups in Thailand (red) and other modern populations in East and Southeast Asia. Reference populations are labeled with different colors based on their language family. The TK populations are separated by ethnicity, while other ethnolinguistic groups are grouped according to their language family. Fig. S10 Average pairwise identity-by-descent (IBD) sharing between populations. Each heatmap panel represents the average total IBD segment length shared between individuals from each population pair, summarized across different segment length categories: (top) all detected IBD segments (≥ 1 cM); (middle) segments of 1–5 cM, reflecting more ancient shared ancestry (~ 500–1500 years ago); and (bottom) segments > 5 cM, indicating more recent ancestry, within the last ~ 500 years. Fig. S11 Estimates of effective population sizes for some TK speakers in southern China across the past 150 generations. Fig. S12 Admixture proportions among TK-speaking populations in Thailand were estimated using SOURCEFIND, with ethnic populations outside Thailand as surrogates. Bar plots are colored by TK-speaking surrogates, while ancestry from other language groups is represented by dot plots, as indicated in the key in the right panel. Fig. S13 Manhattan plots of PBS values for the Lue population using Li as the ingroup and Han_Shanxi as the outgroup. The red dashed line marks the top 0.1% threshold. SNPs above this threshold are shown in black, others in gray. Red dots represent SNPs shared between the Lue population and each TK group in southern China across six panels: (A) Dai, (B) Gelao, (C) Zhuang, (D) Mulam, (E) Dong, and (F) Maonan

## Data Availability

All data generated or analysed during this study are included in this published article, its supplementary information files, and publicly available repositories. The datasets generated and analysed during the current study are available in the Zenodo repository (10.5281/zenodo.14958050). Data are made available upon the electronic request to the corresponding authors confirming that the data will only be used in accordance with the restrictions of the informed consent, including the following: the data will not be transferred to anyone else; the data will be used only for genetic/anthropological studies but not for health or disease-related studies or for any commercial purpose; and no attempt will be made to identify any of the sample donors. Previously published datasets analyzed in this study are available in public repositories, including the Allen Ancient DNA Resource (AADR) (https://dataverse.harvard.edu/dataset.xhtml?persistentId = doi:10.7910/DVN/FFIDCW) and Kampuansai et al. (2023) (https://zenodo.org/records/8129076). The datasets of Liu et al. (2020) and Kutanan et al. (2021) were obtained through personal request.

## Abbreviations

AADR: Allen Ancient DNA Resource
AA: Austroasiatic
AN: Austronesian
BA: Bronze Age
CNG: China Guangxi
CN: China
EM: Expectation maximization
GMS: Greater Mekong Subregion
HB: Historical/Burial cave
HM: Hmong-Mien
IA: Iron Age
IBD: Identity-by-descent
LA: Laos
MAF: Minor allele frequency
MY: Malaysia
N: Neolithic
N.BA: Neolithic to Bronze Age
NJ: Neighbor-Joining
PBS: Population branch statistics
PCA: Principal component analysis
SNP: Single-nucleotide polymorphism
ST: Sino-Tibetan
TH: Thailand
TK: Tai-Kadai
TW: Taiwan
VN: Vietnam
