# Supplementary material for: Genomic continuity of Tai-Kadai-speaking populations from Southern China to Northern Thailand
Source: BMC Biol. 2025 Dec 22;23:361. doi: 10.1186/s12915-025-02467-6 (PMC12723852; doi:10.1186/s12915-025-02467-6)
Supplement: Supplementary file 2 — Additional file 2: Fig. S1 The mean cross-validation values of 10 run ADMIXTURE ranging from K = 2 to 13. Fig. S2 Unsupervised ADMIXTURE diagram illustrates the genetic components of modern and ancient populations in South Asia, Northeast Asia, and Southeast Asia, delineated into K = 10 groups of ancestral components with AncestryPainter. Each individual is depicted by a bar segmented into K colored sections, representing their estimated ancestry components. Populations are demarcated by black lines, and their linguistic family is labeled outside the diagram. Newly generated samples are labeled in red by their population names. [Abbreviation for ancient samples: Ho = Hoabinhian, P = Paleolithic, M = Mesolithic, N = Neolithic, IA = Iron Age, BA = Bronze Age, HB = Historical/Burial caves, TW = Taiwan, CN = China, CNG = China Guangxi, LA = Laos, TH = Thailand, VN = Vietnam, ID = Indonesia, MY = Malaysia, MN = Mongolia, RU = Russia, SA = South Africa, JP = Japan, US = United States; Suffix abbreviation for TK samples in Thailand: N = Northern, NE = Northeastern, C = Central, and S = Southern]. Fig. S3 ADMIXTURE diagram showing the genetic components of various East and South Asian populations. K values ranging from 2 to 13 divided into groups from K = 2 to K = 13 using PONG program. Each individual is represented by a bar divided into K colored segments, indicating their estimated membership fractions in each of the K ancestry component. Fig. S4 Quantitative measurement for pairwise genetic affinity based on allele sharing. Outgroup-f3 in the form f3(Mbuti; X, Y) measuring shared genetic drift between pairwise modern populations. Fig. S5 Quantitative measurement of genetic affinity between pairwise TK-speaking populations based on outgroup-f3 in the form f3(X, Y; Mbuti). Fig. S6 Geographic distribution of genetic affinity between TK populations in Thailand and Southern China, visualized by color-scaled outgroup f3 values in the form f3(Thailand_TK, Southern_China_TK; Mb [file 12915_2025_2467_MOESM2_ESM.pdf]

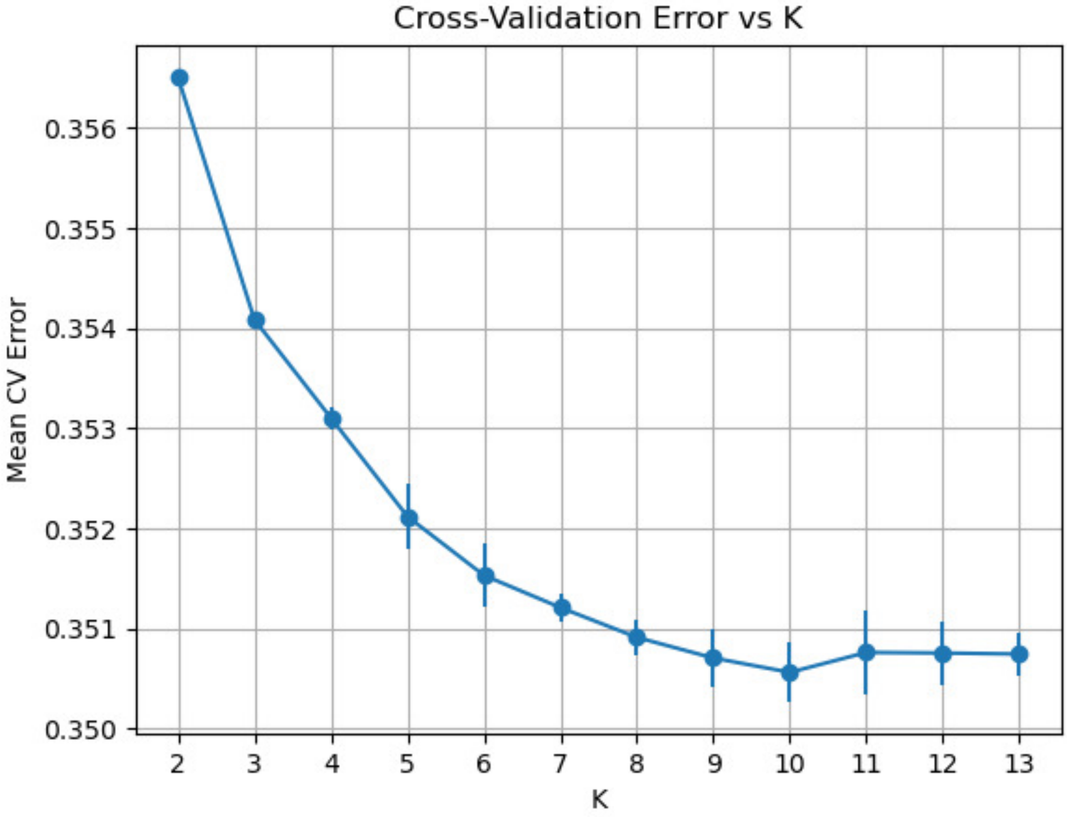

Fig. S1 The mean cross-validation values of 10 run ADMIXTURE ranging from K=2 to 13.

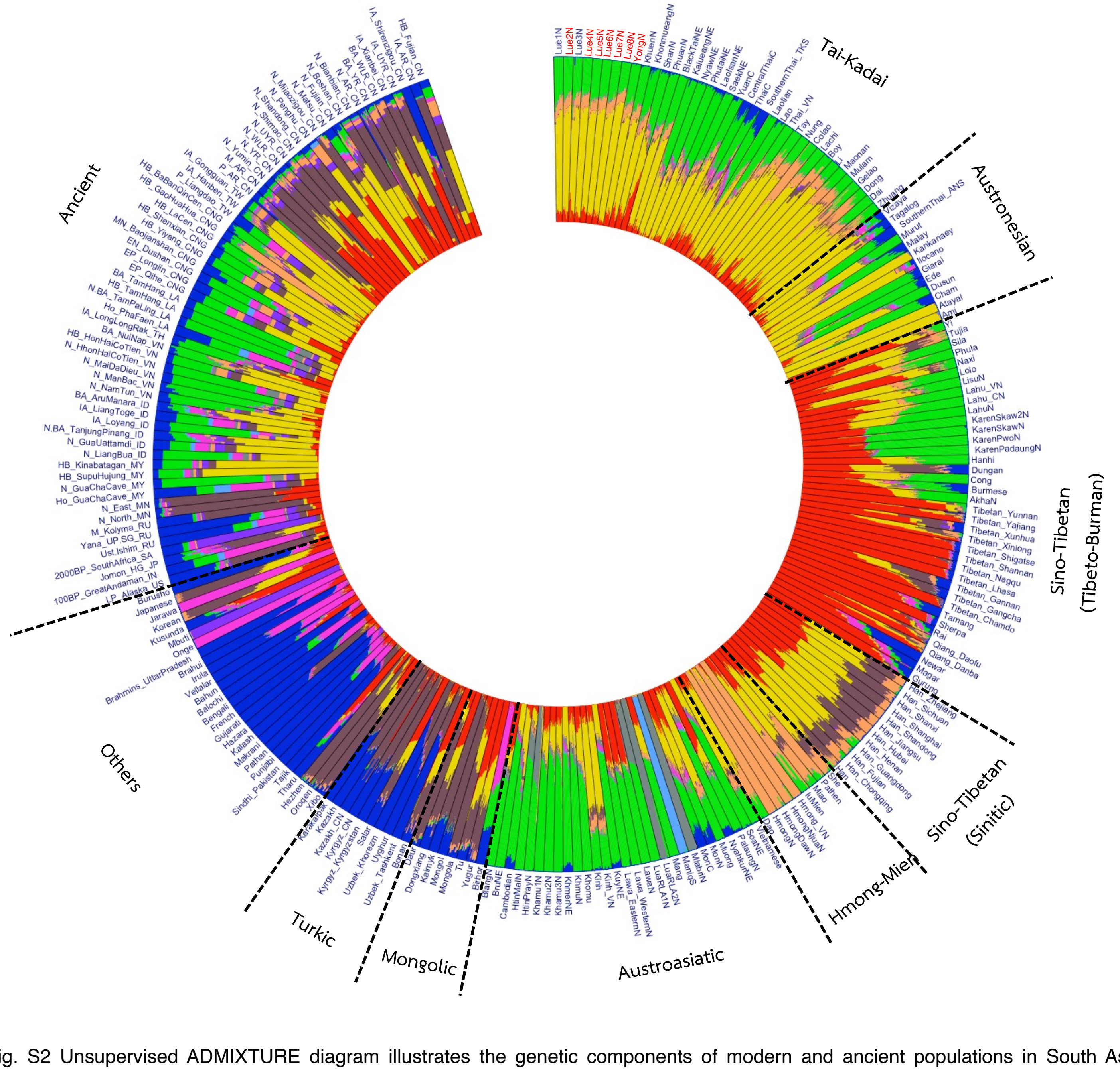

Fig. S2 Unsupervised ADMIXTURE diagram illustrates the genetic components of modern and ancient populations in South Asia, Northeast Asia, and Southeast Asia, delineated into K=10 groups of ancestral components with Ancestrypainter. Each individual is depicted by a bar segmented into K colored sections, representing their estimated ancestry components. Populations are demarcated by black lines, and their linguistic family is labeled outside the diagram. Newly generated samples are labeled in red by their population names. [Abbreviation for ancient samples: Ho = Hoabinhian, P = Paleolithic, M = Mesolithic, N = Neolithic, IA = Iron Age, BA = Bronze Age, HB = Historical/Burial caves, TW = Taiwan, CN=China, CNG = China Guangxi, LA = Laos, TH = Thailand, VN = Vietnam, ID = Indonesia, MY = Malaysia, MN = Mongolia, RU = Russia, SA = South Africa, JP = Japan, US = United States; Suffix abbreviation for TK samples in Thailand: N = Northern, NE = Northeastern, C = Central, and S = Southern].

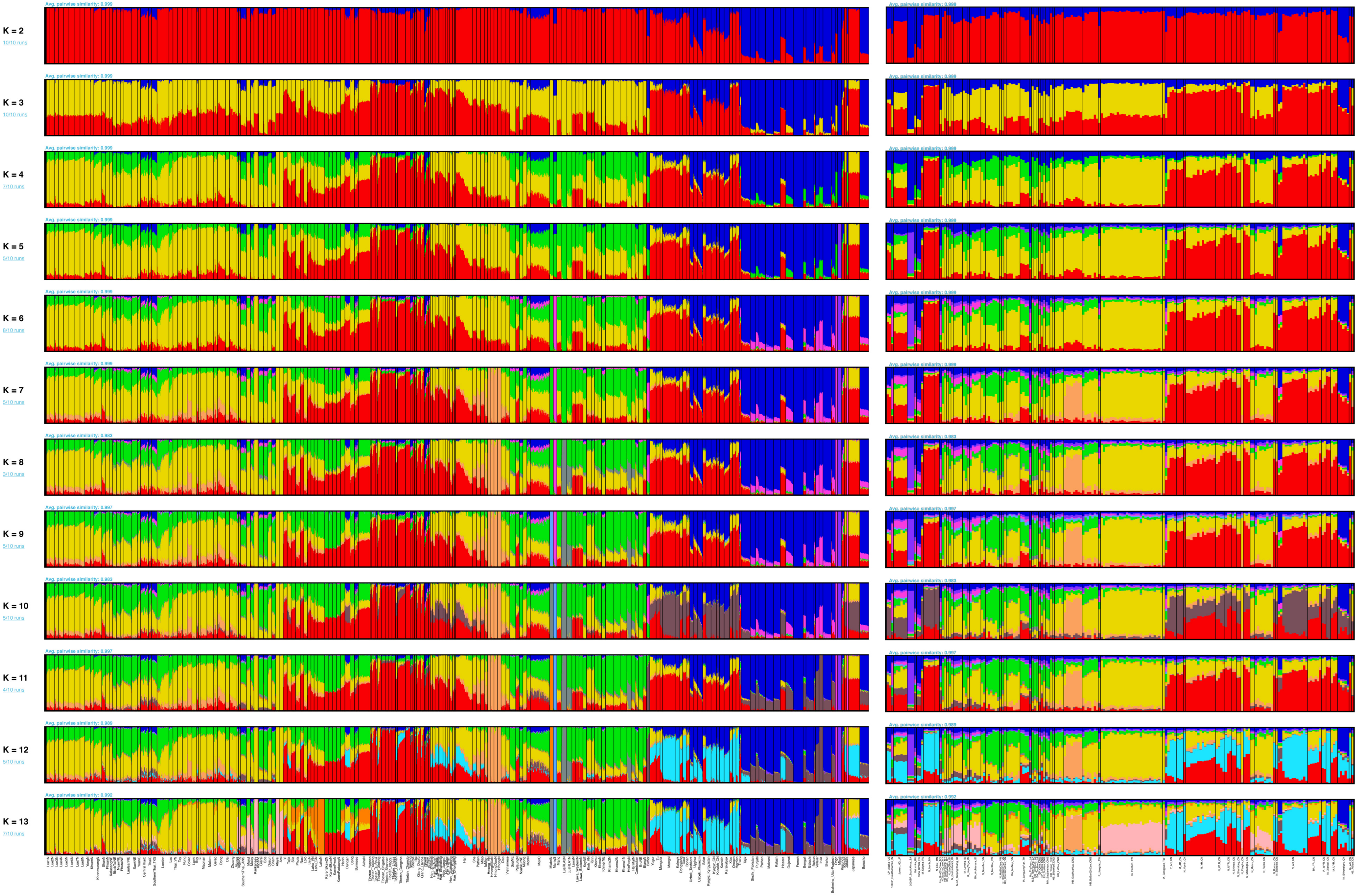

Fig. S3 ADMIXTURE diagram showing the genetic components of various East and South Asian populations. K values ranging from 2 to 13 divided into groups from K=2 to K=13 using *pong* program. Each individual is represented by a bar divided into K colored segments, indicating their estimated membership fractions in each of the K ancestry component.

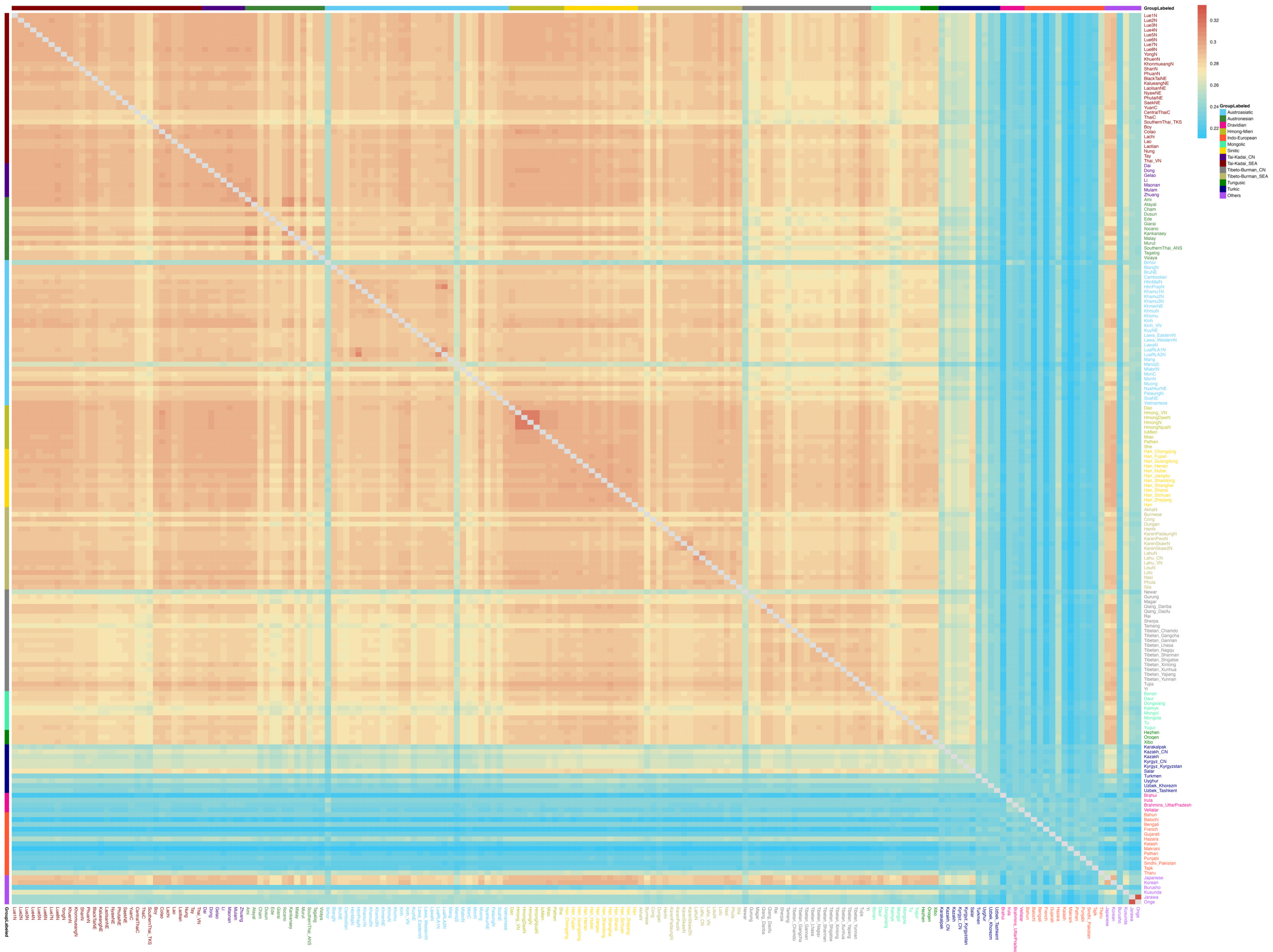

Fig. S4 Quantitative measurement for pairwise genetic affinity based on allele sharing. Outgroup-f_3 in the form f_3 (Mbuti; X, Y) measuring shared genetic drift between pairwise modern populations.

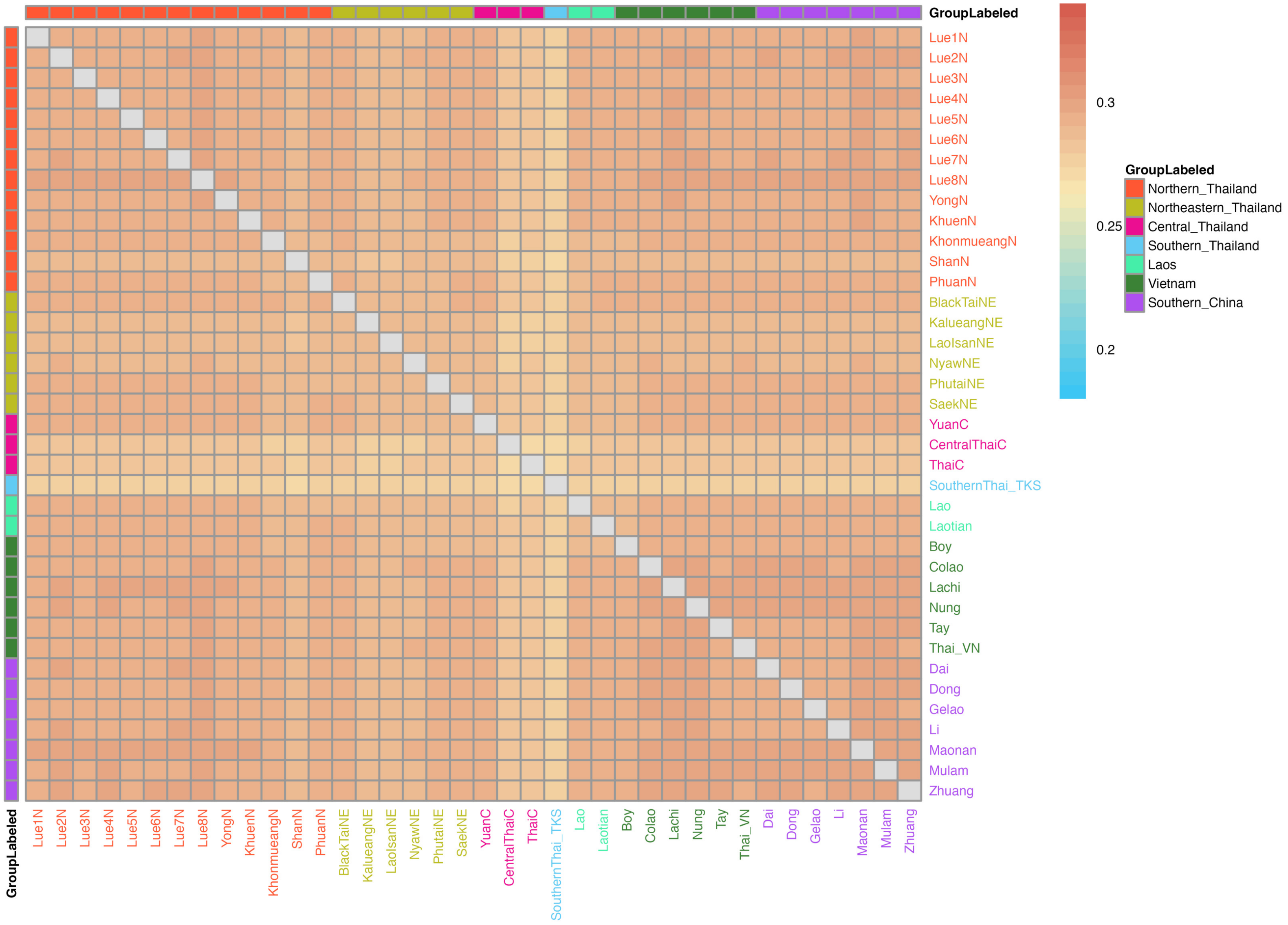

Fig. S5 Quantitative measurement of genetic affinity between pairwise TK-speaking populations based on outgroup-f_3 in the form f_3(X, Y; Mbuti).

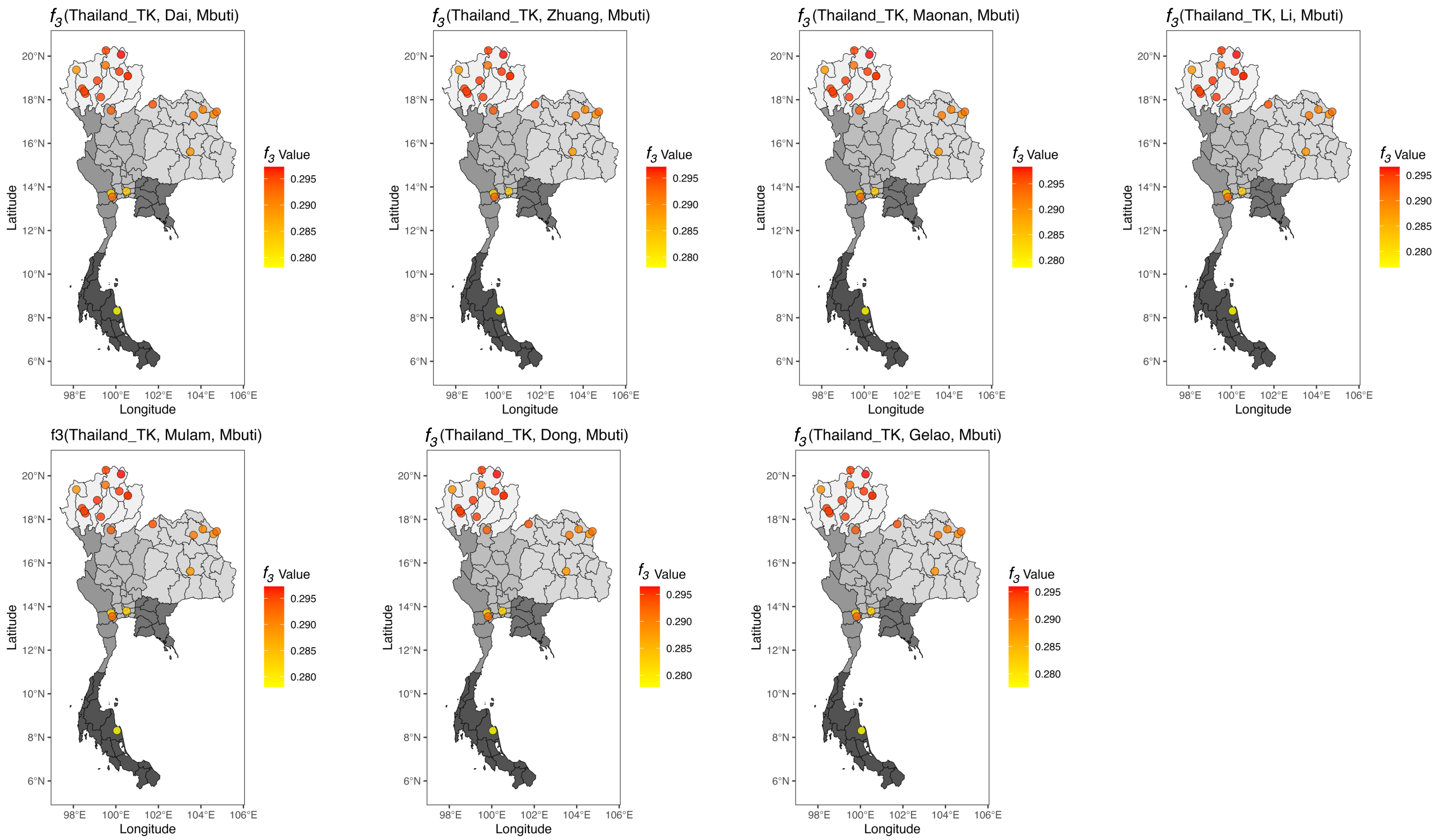

Fig. S6 Geographic distribution of genetic affinity between TK populations in Thailand and Southern China, visualized by color-scaled outgroup f_3 values in the form f_3(Thailand_TK, Southern_China_TK; Mbuti). Population locations are plotted according to geographic coordinates, with warmer colors indicating higher allele sharing.

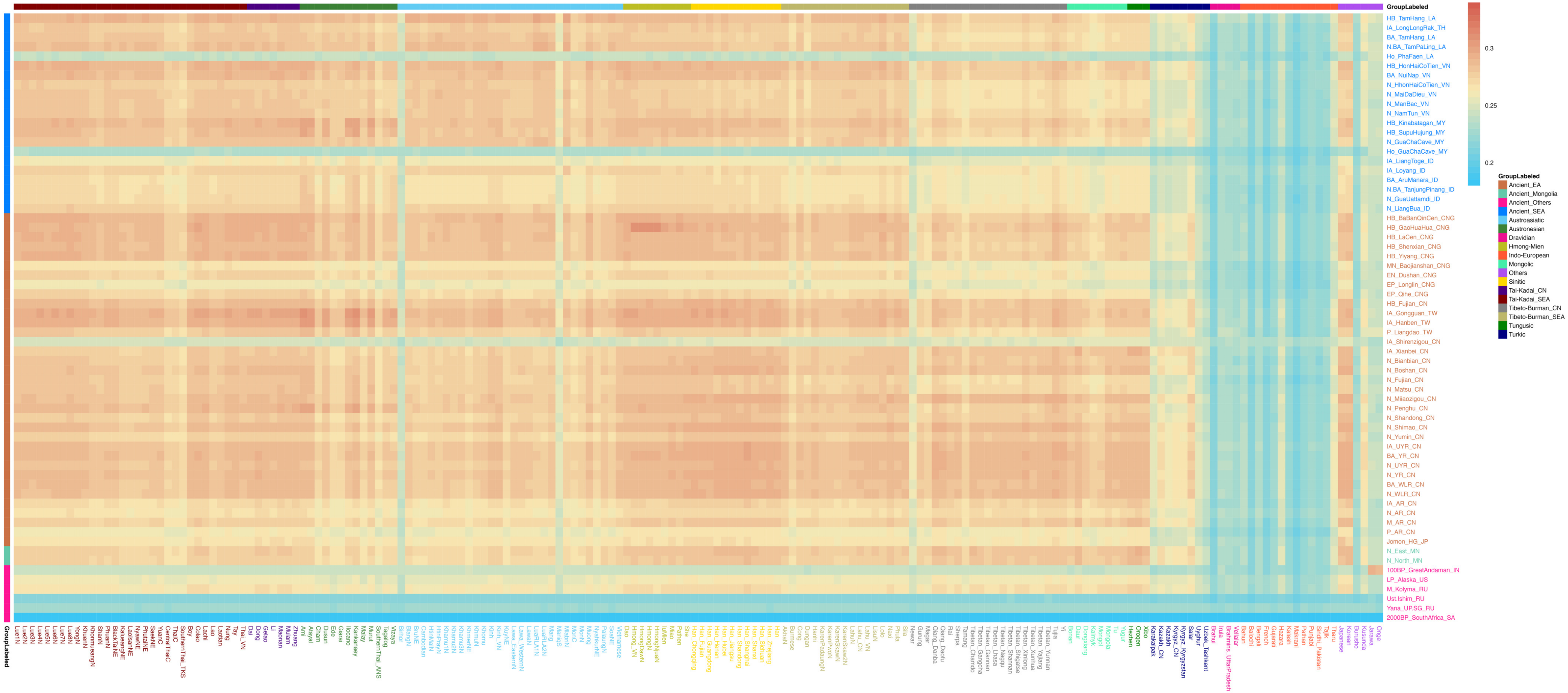

Fig. S7 Quantitative measurement for pairwise genetic affinity based on allele sharing. Outgroup- f_3 in the form f_3(Mbuti; X, Y) measuring shared genetic drift between pairwise modern and ancient populations.

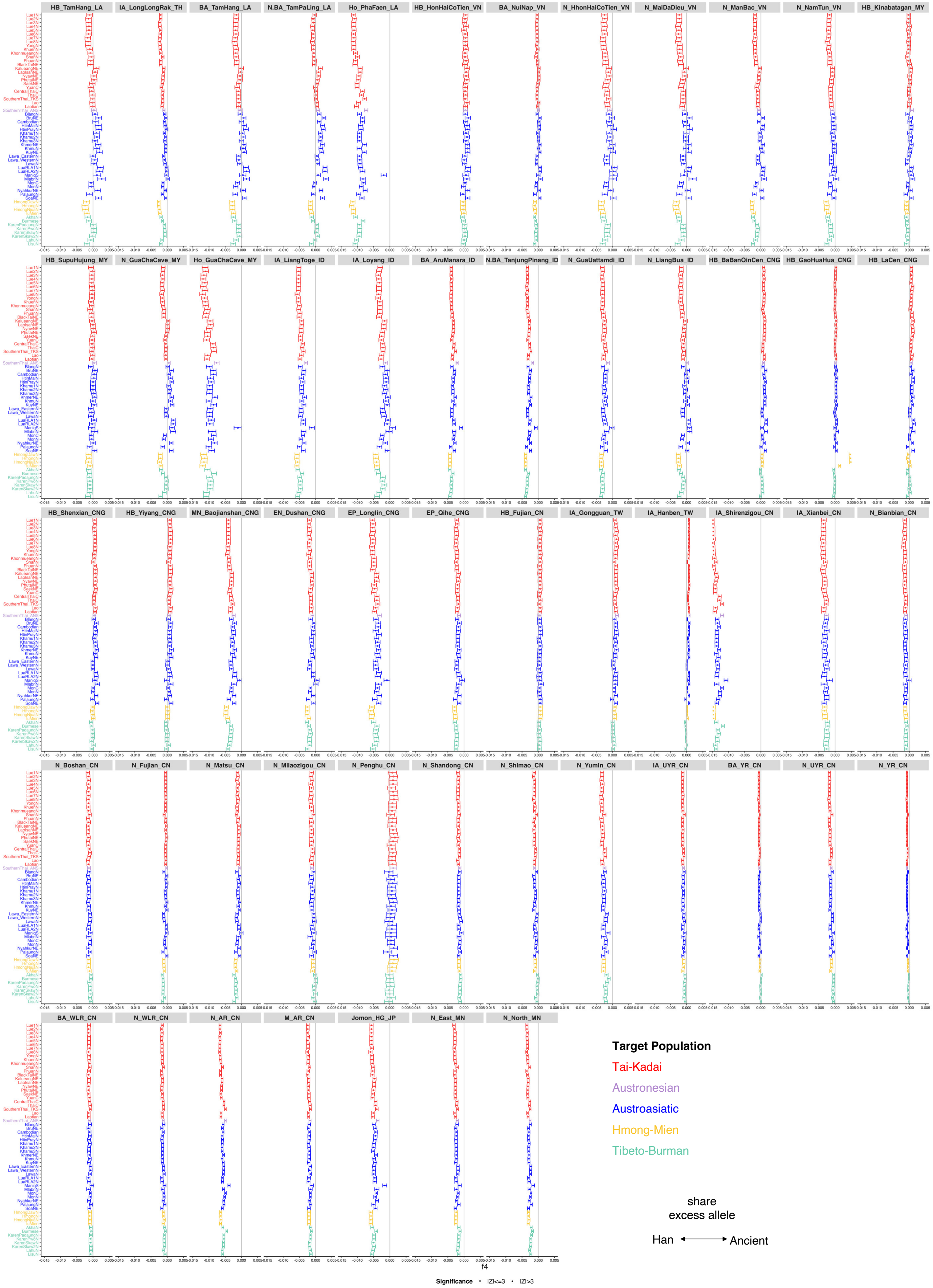

Fig. S8 f_4 statistics comparing TK-speaking ethnic groups in Thailand and neighboring countries to the ancient DNA from the Yellow River to Mekong regions. Z-scores are for f_4(Ancient sample, Han Chinese; Target, French), where the target is the Southeast Asian ethnic group. The vertical grey lines denote 0. Ethnic names are colored according to language family. Empty circles denote nonsignificant Z-scores (|Z|≤3), while solid circles denote significant Z-scores (|Z|>3).

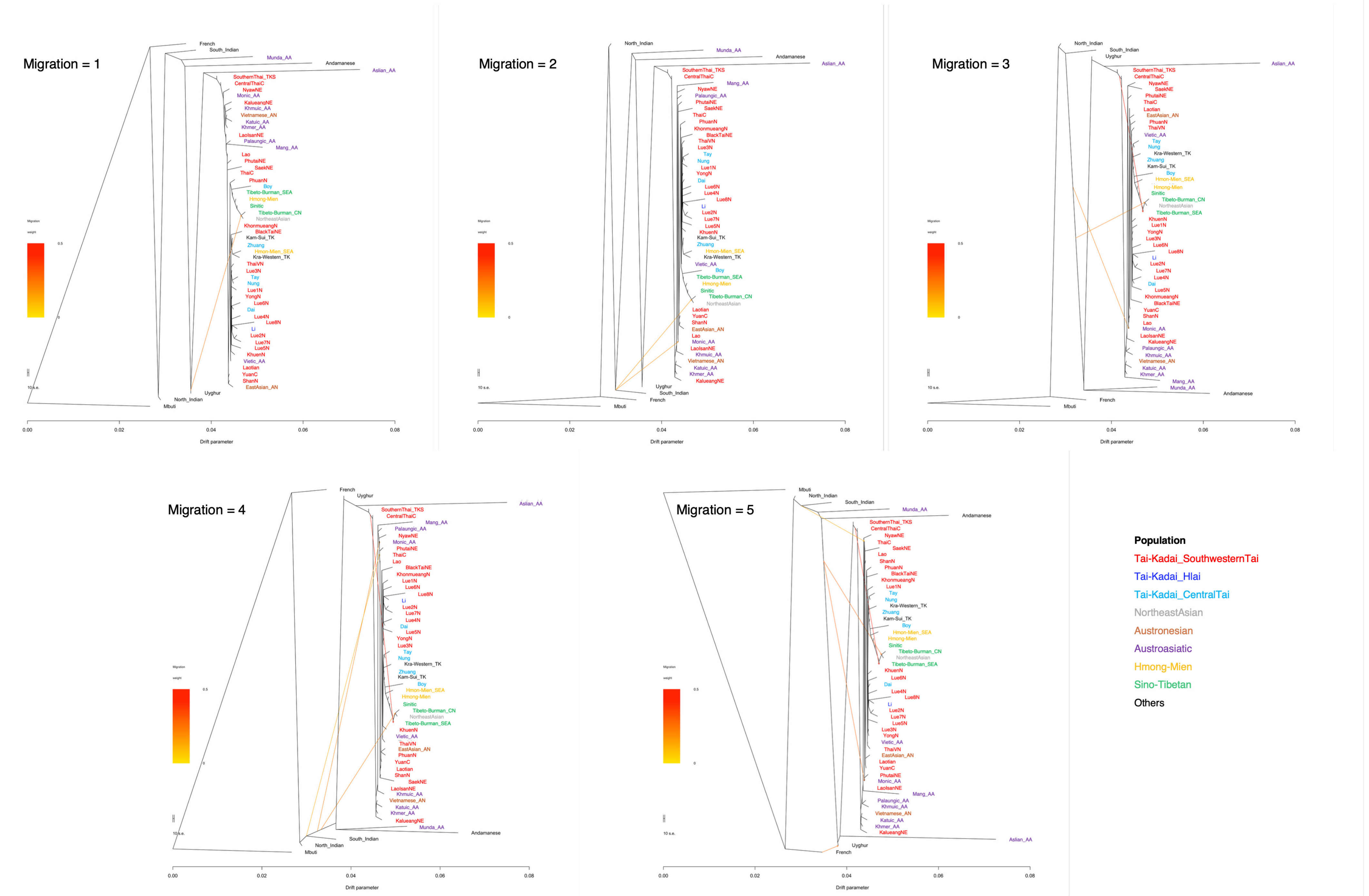

Fig. S9 TreeMix diagram with 1-5 migration events for the TK-speaking ethnic groups in Thailand (red) and other modern populations in East and Southeast Asia. Reference populations are labeled with different colors based on their language family. The TK populations are separated by ethnicity, while other ethnolinguistic groups are grouped according to their language family.

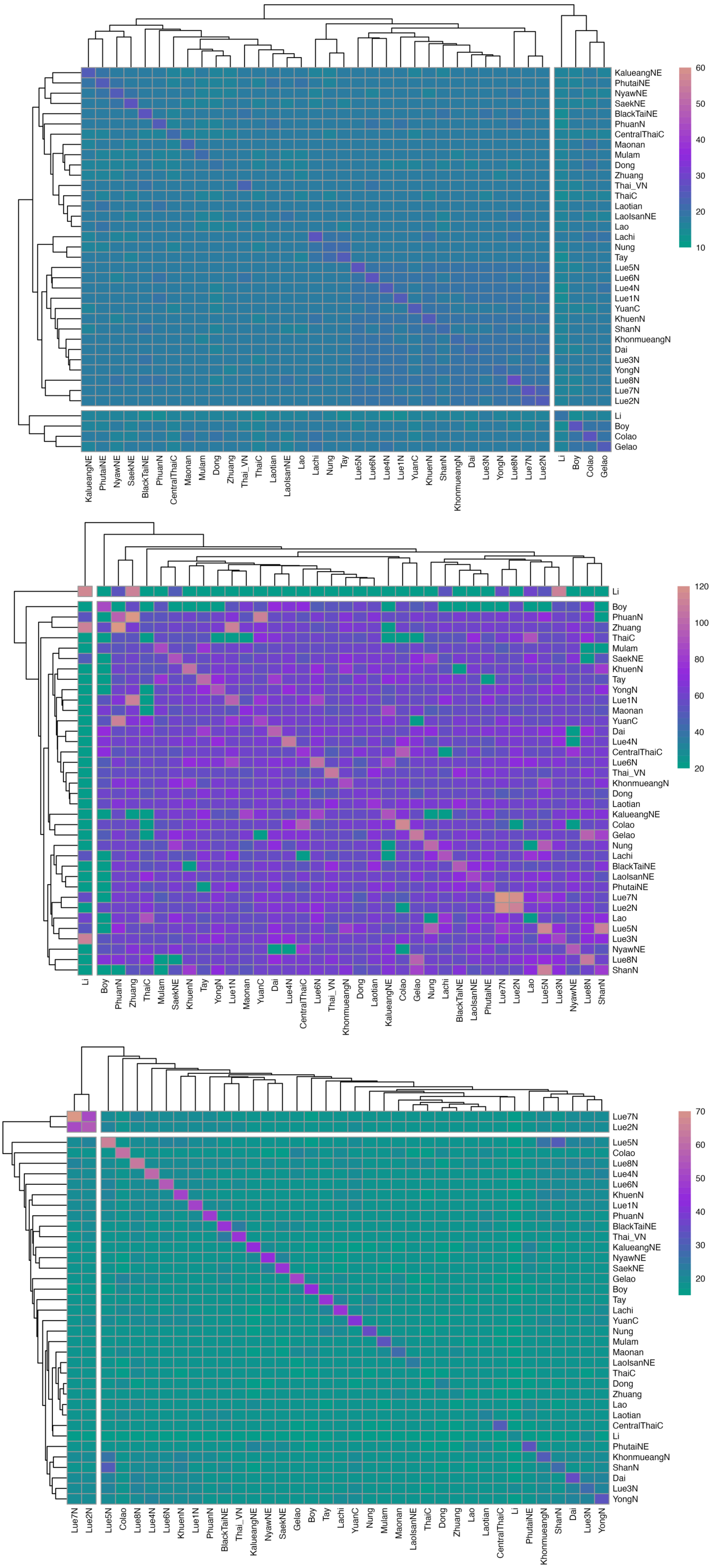

Fig. S10 Average pairwise identity-by-descent (IBD) sharing between populations. Each heatmap panel represents the average total IBD segment length shared between individuals from each population pair, summarized across different segment length categories: (top) all detected IBD segments (≥1 cM); (middle) segments of 1–5 cM, reflecting more ancient shared ancestry (~500–1500 years ago); and (bottom) segments >5 cM, indicating more recent ancestry, within the last ~500 years.

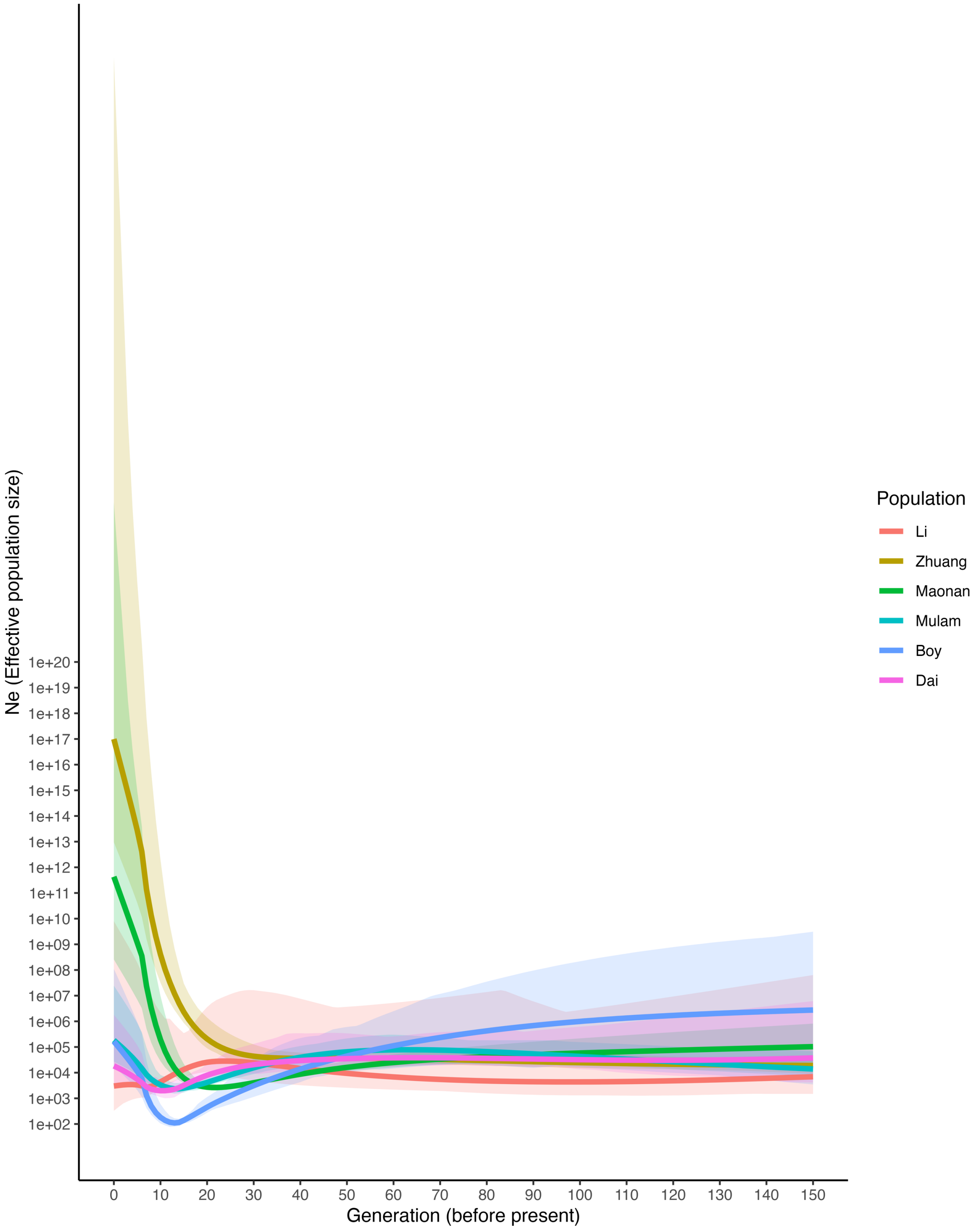

Fig. S11 Estimates of effective population sizes for some TK speakers in southern China across the past 150 generations.

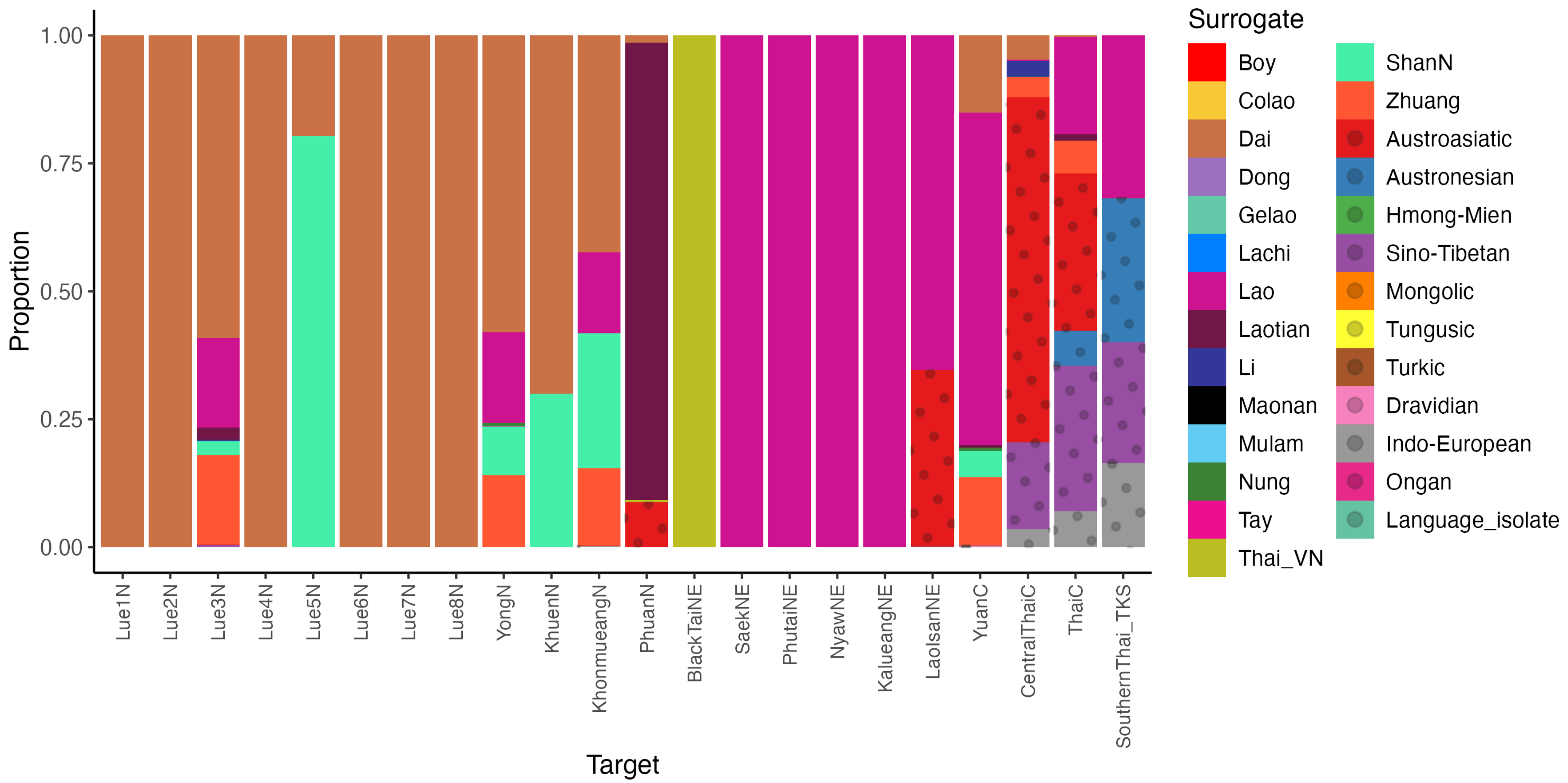

Fig. S12 Admixture proportions among TK-speaking populations in Thailand were estimated using SOURCEFIND, with ethnic populations outside Thailand as surrogates. Bar plots are colored by TK-speaking surrogates, while ancestry from other language groups is represented by dot plots, as indicated in the key in the right panel.

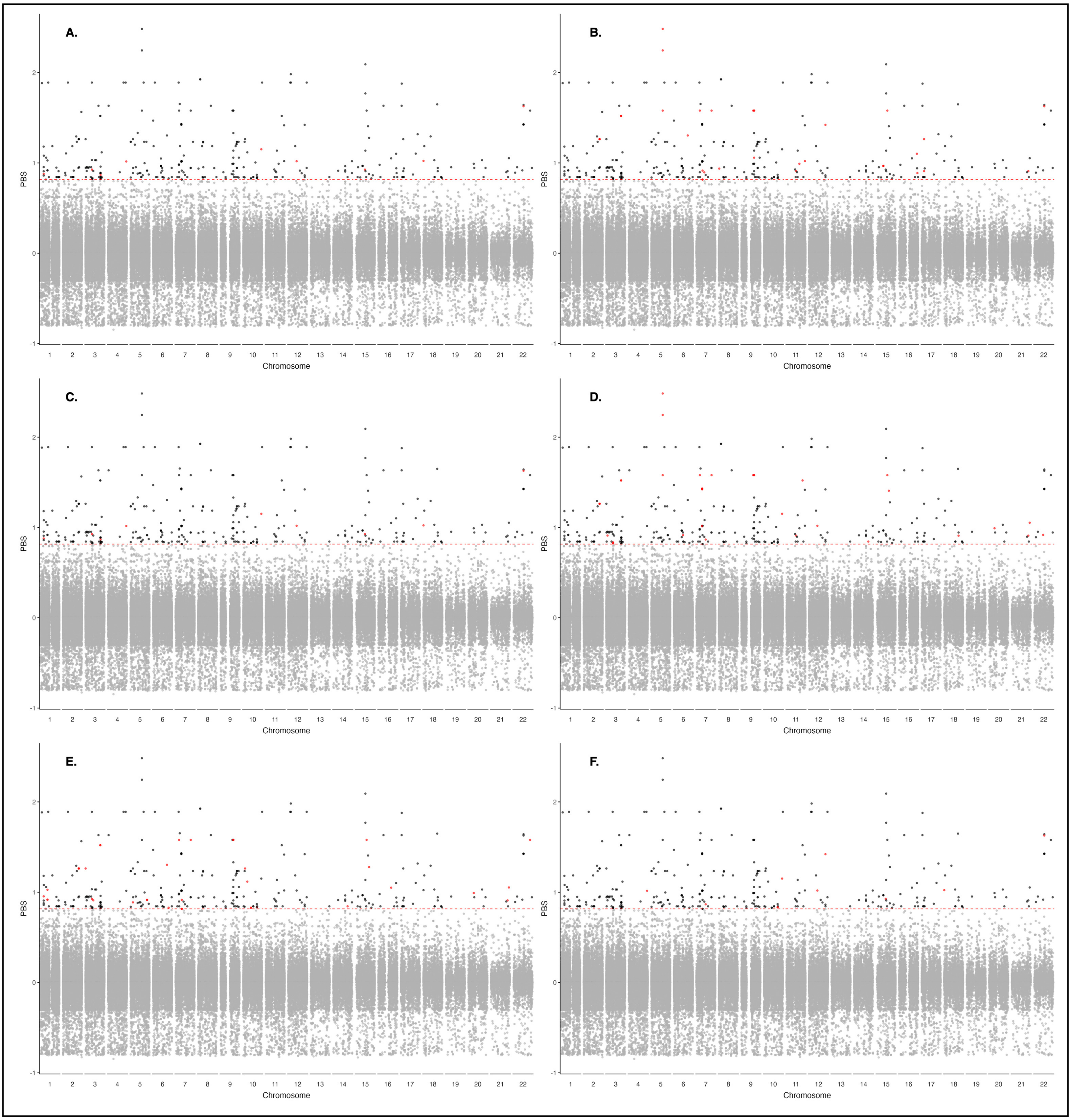

Fig. S13 Manhattan plots of PBS values for the Lue population using Li as the ingroup and Han_Shanxi as the outgroup. The red dashed line marks the top 0.1% threshold. SNPs above this threshold are shown in black, others in gray. Red dots represent SNPs shared between the Lue population and each TK group in southern China across six panels: (A) Dai, (B) Gelao, (C) Zhuang, (D) Mulam, (E) Dong, and (F) Maonan.
